# Nanoparticles‐induced potential toxicity on human health: Applications, toxicity mechanisms, and evaluation models

**DOI:** 10.1002/mco2.327

**Published:** 2023-07-14

**Authors:** Lihui Xuan, Zhao Ju, Magdalena Skonieczna, Ping‐Kun Zhou, Ruixue Huang

**Affiliations:** ^1^ Department of Occupational and Environmental Health Xiangya School of Public Health Central South University Changsha Hunan China; ^2^ Department of Systems Biology and Engineering Institute of Automatic Control Silesian University of Technology Gliwice Poland; ^3^ Biotechnology Centre, Silesian University of Technology Gliwice Poland; ^4^ Beijing Key Laboratory for Radiobiology Department of Radiation Biology Beijing Institute of Radiation Medicine Beijing China

**Keywords:** evaluation technique, health application, mechanism of toxicity, nanoplastics

## Abstract

Nanoparticles (NPs) have become one of the most popular objects of scientific study during the past decades. However, despite wealth of study reports, still there is a gap, particularly in health toxicology studies, underlying mechanisms, and related evaluation models to deeply understanding the NPs risk effects. In this review, we first present a comprehensive landscape of the applications of NPs on health, especially addressing the role of NPs in medical diagnosis, therapy. Then, the toxicity of NPs on health systems is introduced. We describe in detail the effects of NPs on various systems, including respiratory, nervous, endocrine, immune, and reproductive systems, and the carcinogenicity of NPs. Furthermore, we unravels the underlying mechanisms of NPs including ROS accumulation, mitochondrial damage, inflammatory reaction, apoptosis, DNA damage, cell cycle, and epigenetic regulation. In addition, the classical study models such as cell lines and mice and the emerging models such as 3D organoids used for evaluating the toxicity or scientific study are both introduced. Overall, this review presents a critical summary and evaluation of the state of understanding of NPs, giving readers more better understanding of the NPs toxicology to remedy key gaps in knowledge and techniques.

## Graphical abstract

A comprehensive landscape of the applications of NPs and their toxicity on health.

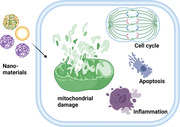

## 1 INTRODUCTION

Nano is the abbreviation for milli‐micron, one of the units of length. A nanometer is one billionth of a meter, which is so small that it is equivalent to the length of ten hydrogen atoms lined up in a row.^1^ The current consensus is that the size range for nanoparticles (NPs) is 1–100 nm, to avoid referring to clusters of atoms as particles, particles smaller than 1 nm are excluded from NPs, but there is literature where NPs include particles smaller than 1 nm.^2^, ^3^ Nanomaterials are materials with at least one dimension (length, width, or height) between 1 and 100 nm. They can be made from a variety of materials, including metals, semiconductors, ceramics, and polymers, and they have unique physical and chemical properties that differ from their bulk counterparts. The small size provides many properties for NPs such that there are more applications. The small size and inhomogeneous electron distribution of NPs allow for a wider range of applications for their magnetic properties and those of their suspensions,^4^ such as data storage,^5^ drug transport,^6^ environmental purification,^7^ and so on. NP properties include not only a small size but also a large surface area, which facilitates their interaction with molecules at the target site and mediates a range of toxicity mechanisms. The property of NPs correlates well with organism response severity and toxicity. Compared with larger NPs, the smallest (10 nm) NPs improve silver (Ag) tissue distribution and increase hepatobiliary toxicity.^8^ And smaller NPs are more likely to be taken up by cells and cause cytotoxicity.^9^, ^10^, ^11^ In addition, the distribution and accumulation of NPs at the target site and the interaction with other molecules depend on their surface charge and aggregation state. Due to their small size, NPs also have special effects such as the small size effect, special magnetic properties, the quantum size effect, and the macroscopic quantum tunneling effect, which are not found in macroscopic particles.^12^, ^13^ All the characteristics of NPs are the reason why NPs have been widely used in a variety of fields.^14^

The explosion in nanomaterials research has fueled the advancement of nanotechnology. Nanotechnology is a means of studying and exploiting matter and the structure of matter at the nanometer scale. Nanotechnology developed exponentially in the 1980s with the advent of scanning tunneling microscopy, a technique with atomic resolution.^15^, ^16^ And other equipment such as scanning transmission electron microscopy and tandem electron microscopy, combined with precise procedures such as electron beam micrography, allow us to operate with precision and produce nanostructured materials, while research related to nanotechnology is growing exponentially.^17^, ^18^ Nanotechnology can be applied to produce new functional electronic devices with higher speed and lower energy consumption,^19^ invent new drugs,^20^ and even to duce new materials for food testing and pollution monitoring.^21^, ^22^, ^23^ In general, with the help of nanotechnology and NPs, we can prepare materials that are smaller in size and have the same or even better properties, reducing the size and weight of objects. Computers were miniaturized and made popular with the help of micron‐level semiconductor manufacturing technology.^24^ The advantages of “miniaturization” are astounding, both in terms of energy and resource use. Nanotechnology, on the other hand, allows nanomaterials to be expected to have higher optical, electrical, magnetic, and thermal properties, as previously mentioned,^25^, ^26^ and involves mechanical properties such as increased strength and toughness.

With the rapid development of nanotechnology, NPs are widely used. On the one hand, nanotechnology provides a new platform for advances in industry, agriculture, and medicine,^27^, ^28^ which plays a key role in promoting global economic growth,^29^, ^30^ and a wide range of nanoscale materials with a variety of functions are being produced at a rapid rate for academic and industrial purposes.^31^, ^32^, ^33^ On the other hand, the prospect of nanotechnology lies in the rational use of nanomaterials and minimizing the harmful effects on human health, the environment, and society.^34^ However, there is already a substantial amount of information on human and environmental hazards and exposures during the production, processing, using and recycling of NPs, including increased and prolonged exposure of production workers to NPs, increased environmental exposure of the local population of the manufacturing plant, as well as of consumers who use products containing NPs, and the hazards of NPs that may escape from waste treatment facilities. Some of the previously mentioned NPs have unprecedented properties, the normal defense mechanisms of the human body may not be able to deal with them adequately.^35^ NP contamination typically results in lung effects and triggers an inflammatory response, but the severity of the response is not fully understood in relation to the type and concentration of NPs, and the health damage caused by long‐term or repeated exposure is unknown.^36^ Another noteworthy aspect of the rise of nanotechnology in medicine is that, while NPs play a beneficial role, they may also have some negative health effects, but the exact health effects are unknown.^37^

The extent to which the health risks of NPs, their toxicokinetic in humans and the environment, differences in their distribution in different air, terrestrial and aquatic environments, and effective mechanisms for their degradation can be predicted from the available data is not yet clear and sufficient. As the ecotoxic effects of NPs and the risks of nanotechnology have attracted attention, it has become an inescapable prerequisite for the development of nanotechnology to organize interdisciplinary cooperation in the face of social needs, and to develop appropriate risk statute theories as soon as possible to introduce effective preventive and regulatory measures before the arrival of nanotechnology hazards, minimize the social risks of nanotechnology and increase the sustainability of nanotechnology development. Therefore, a comprehensive re‐examination of NPs and nanotechnology is needed for more accurate utilization and sustainable development. In this review, we enumerate the applications of nanotechnology related to human health, including industrial, agricultural, and medical aspects. As well as the specific toxicity on various health systems and the biological mechanisms for human health. Finally, current 2D, 3D or emerging models for evaluating NPs are summarized. It can be seen that nanomaterials are involved in various industries and are relevant to health.

## 2 NANOTECHNOLOGY APPLICATION IN HEALTH

With the continuous progress of science and technology and the rapid development of nanotechnology, NPs are being used more and more widely in various fields, including but not limited to medical, agricultural, environmental, energy and material science. The properties and behavior of matter frequently change dramatically as we scale down from the macroscopic scale to the micrometer and then to the nanometer. Because of this change in properties, NPs and nanotechnology are extremely important for various applications and are highly relevant to health^38^: in environmental remediation, NPs can be used to remove pollutants from soil and water; in food and beverage, NPs can be used as food additives to enhance flavor, texture, and stability; in cancer therapy, NPs can be used to deliver chemotherapy drugs directly to cancer cells, improving drug efficacy and reducing side effects. However, this wide application also brings some potential risks and safety issues that need attention and concern. And it is important to note that the safety and long‐term effects of these applications on human health and the environment are still being studied and evaluated. Nanotechnology is already being used in a variety of industries and is actively being developed, in this section, we review the industrial, agricultural and medical applications of nanotechnology in relation to human health.

### 2.1 Nanotechnology and industrial

One of the research objectives of nanotechnology as an interdisciplinary and innovative technology is to produce products that meet the needs of industrial practice and thus provide convenience to people's lives. This section provides a brief overview of nanotechnology and those applications in industry that are relevant to human health.

Nanotechnology is already being used in the production of consumer goods such as textiles,^39^ paints, and cosmetics,^40^ as well as allowing for smaller, faster, and less consuming electronic devices. In environmental protection, nanotechnology can be used to treat environmental pollution, such as nanocatalysts can be used to purify air or water, nanosorbent materials can be used to treat wastewater or sludge, nanophotocatalytic materials can be used to degrade organic pollutants, and so on. A environmental research indicated cadmium sulfide (CdS‐NPs) attached to the surface of NiO crystal plates caused a change in the energy band structure, generating more free electrons on the CdS surface. The material was added as a powder to Congo Red dye effluent and was able to significantly increase the decomposition efficiency of organic pollutants under visible light irradiation.^41^ Nanoceramics provide another example, hard, wear resistant, high temperature resistant, and corrosion resistant. Ceramic products spiked with zinc oxide (ZnO)‐NPs have an antibacterial and deodorizing effect and a self‐cleaning effect that decomposes organic matter, greatly improving product quality.^42^ The application of nanotechnology in the development of functional textile fabrics has also yielded some results, such as the addition of NPs such as TiO₂, ZnO, and SiO₂ to chemical fibers leading to ultraviolet (UV) rays absorption, which can effectively protect the human body from UV damage. The addition of NPs with semiconductor properties such as TiO₂‐NPs can reduce the electrostatic effect.^39^ The application of nano‐nickel oxide can create radiation‐protective fabrics. Some NPs such as nano‐zirconia have strong absorption properties in the mid‐infrared band and can be used to make far‐infrared functional fabrics.^43^ Nanotechnology can also be used to make new types of drugs and medical devices, such as nanomedicines, nanoprobes, nanosensors. These technologies can improve the efficiency of drugs and reduce side effects, as well as be used for early diagnosis and treatment of diseases.

However, in industrial processes, especially those involving high energy or pressure technologies, NP leakage is more likely to occur, causing environmental contamination, and the enhanced diffusivity significantly increases the risk of dust explosions. In summary, information is needed on the bioaccumulation of NPs and the potentially toxic effects of inhalation and ingestion of NPs, and their long‐term effects on public health. The environmental consequences of the eventual disposal of these materials also need to be carefully evaluated.

### 2.2 Nanotechnology and agriculture

Nanotechnology has shown commendable potential for improving planting and farming methods, optimizing the processing and packaging of products, improving the agricultural environment, and facilitating the development of genetically modified technology.^44^ The disadvantage is that due to the small size, as well as the larger surface/mass ratio properties, NPs have a greatly enhanced ability to penetrate biological membranes and a higher risk of accumulation in various organisms. This section is an exploration of the impact nanotechnology has on agriculture.^45^, ^46^

The health‐related aspects of nanotechnology applications in agriculture involve food safety and ecological protection. Due to the limitations of traditional pesticides in terms of rich organic solvents, poor dispersion, and easy dust drift, most pesticides are not fully effective and cause environmental pollution. The application of nanotechnology and NPs can change the physical and chemical properties of pesticides into stable homogeneous bodies that are highly dispersed and easily suspended in water, which can fully improve the utilization rate of pesticides, reduce pesticide residues and reduce environmental pollution.^47^ Nanotechnology can also be used as a plant growth regulator, which can enhance the activity of seeds,^48^ promote the growth of plant roots, and further improve the resistance to insects, diseases, and various kinds of the resilience based on the original, to achieve the effect of increasing yield and quality improvement.^49^ For example, multiwalled carbon nanotubes (CNTs) can significantly increase the germination rate and root extension of vegetable seeds, and the photochemical effect of TiO_2_ can produce superoxide compounds of reactive oxygen species (ROS),^50^ increasing the resistance of seeds. It is worth noting that the promotion of plant growth by nanomaterials is not unlimited, and that overdoses of the nanomaterials Zn‐NP and ZnO‐NPs can also affect root growth in crops.^51^ These products are more efficient and use less, reducing environmental pollution and the impact on human health. Nanogene vectors^52^ have attracted increasing attention in animal and plant breeding in recent years due to their protective effect on exogenous genes, high penetration, high transfection efficiency, safety, low toxicity, and nonimmunogenicity.^53^ We envisage that the use of nanotechnology to improve feed can significantly increase the utilization of nutrients, as well as adsorb toxic substances and improve the safety and quality of livestock and poultry products. At this stage, however, the safety of nanofeeds and additives is not sufficiently studied, so the development of nanofeeds and additives must be accompanied by a corresponding safety evaluation.^54^ Nanotechnology has also been used in the food industry,^55^ mainly in food packaging where nanotechnology is used to improve food quality and extend shelf life^56^, ^57^ and reduce the breeding of bacteria. The sufficiently large surface area of NPs enhances the interaction of the raw material polymer, which greatly improves the mechanical properties, barrier properties, and thermal stability of the raw material. Nanotechnology can be used to control agricultural pollution and protect ecological environment, such as nano adsorbent can be used to purify soil and water, nano catalyst can be used to treat agricultural wastewater. CNTs have been increasingly used for the adsorption of organic pollutants in wastewater, and nano‐zero‐valent iron can remediate organically contaminated soils.^58^ Nanomaterials are inherently very sensitive chemical and biological sensors, and in combination with technologies such as biochips, nanobiosensors have been used in microbial detection, food detection, and monitoring of metabolites in body fluids.^59^ A team of researchers has developed a gold (Au)/silicon‐based heterogeneous structured nanorod technology for Salmonella detection sensors that can detect the food‐borne pathogen Salmonella in a novel and efficient manner.^60^

Nanotechnology is going to have a huge impact on agriculture in several ways, and it can be beneficial, or it can pose some potential health risks. For example, nanopesticides may affect beneficial insects such as bees, and NPs may enter soil and water bodies, causing potential harm to ecosystems. Therefore, the assessment of the safety of nanotechnology and NPs for human health and the environment should be strengthened,^61^ while the relevant regulatory authorities must also conduct in‐depth research and develop scientific regulatory methods for relevant nanoproducts.^62^, ^63^

### 2.3 Nanotechnology and medical

With a growing number of applications, nanotechnology remains gaining popularity in the medical field.^64^ It uses NPs and nanoscale technology to prevent, diagnose and treat disease through diagnostic tools, delivery systems, and drug treatments.^65^

Efficient and accurate diagnosis is critical in the medical process. While many diagnostic methods detect changes in a biomarker in cells or tissues to indicate the course of a disease, more advanced imaging technologies can detect physiological changes in tissues more visually and also monitor and control the release of drugs, and these higher precision imaging agents have been developed using nanotechnology.^66^, ^67^, ^68^ Iron oxide NPs (Fe_2_O_3_‐NPs or Fe_3_O_4_‐NPs) consist of magnetic hematite (γ‐Fe_2_O_3_) and/or magnetite (Fe_3_O_4_) particles. The prospect of developing a wider range of applications based on Fe_2_O_3_‐NPs due to their improved solubility in organic and aqueous solutions. Iron oxide NPs have been used in various diagnostic and imaging techniques,^69^, ^70^ for example, as contrast agents in magnetic resonance imaging (MRI) or as magnetic sensing probes in vitro diagnostics.^71^, ^72^ Similarly, Au‐NPs are widely used in various optical biosensors and have a promising future in medical diagnostics.^73^ A new fluorescent‐labeled semiconductor quantum dot could also be used as a material for in vivo imaging, an example of using nanomaterials.^74^ These studies have demonstrated the low toxicity and high permeability of nanomaterials, allowing nanotechnology to play an important role in the field of medical diagnostics.

Nanotechnology is thought to be beneficial in preventing and treating cancer and disease.^75^, ^76^, ^77^ In a recent study, the United States Food and Drug Administration‐approved NIR dye ICG was incorporated onto Gd‐DTPA‐human serum albumin (HSA)@ICG‐NPs containing glycyrrhetinic acid‐modified Gd diethylenetriaminepentaacetic acid (Gd‐DTPA) to create the multifunctional nanoreagent Gd‐DTPA‐HSA@ICG‐bevacizumab (NPs‐Bev), a novel multifunctional self‐assembled nanoprobe with high biocompatibility and highly viable therapeutic reagents for MRI, fluorescence imaging and radiotherapy, which is expected to be a multifunctional nanoplatform for breast cancer precision therapy.^78^ A variety of NPs are being tested in preclinical studies for drug and gene delivery, to improve their delivery to specific sites.^79^, ^80^ Here are some of the NPs that have been approved for clinical use, while cancer treatment‐related accounts for a large proportion.^81^ They include liposomal doxorubicin, which is used to treat triple‐negative breast cancer, and non‐small cell lung cancer, which has significantly improved tumor suppression efficiency.^82^ It is worth noting that as one of the most commonly used drug delivery systems, liposomes may be less toxic than other NPs.^83^, ^84^ Gold nanoparticles (Au‐NPs) and silver nanoparticles (Ag‐NPs) are among the metallic NPs that have been studied more intensively. Au‐NPs have negative reactive groups on their surface and are also used in cancer diagnosis and radiotherapy.^85^, ^86^, ^87^ Systems that combine imaging and therapy can even be developed with Au‐NPs.^88^, ^89^ However, there is still a lack of a comprehensive understanding of its toxicity.^90^ The renewed interest in the utility of Ag as a broad‐spectrum antimicrobial agent has led to the development of a variety of products containing Ag‐NPs, such as wound dressings and antimicrobial coatings, to prevent the growth of bacteria on surfaces.^91^ Nanosilver can also be used in biomedical electronics^92^, ^93^, ^94^, ^95^ In addition, CNTs are increasingly being used for biomedical applications such as delivering drugs.^96^, ^97^, ^98^ A study in which nanotechnology is used to deliver thrombin directly to the site of injury using intravenous injection to improve hemostasis.^99^ Improved antimicrobial properties of dental and orthopedic materials in the early stages of implantation by sol–gel implantation of antimicrobial NPs such as Ag–NPs and ZnO–NPs.^100^ The disadvantage of many drugs is their poor solubility in water,^101^ and encapsulating them in NPs using nanotechnology can minimize the need for toxic cosolvents while improving their stability.^102^ NPs can also be used to alter the metabolism of drugs, thereby improving their efficacy.^103^, ^104^ The biological advantages of using nanotechnology to manufacture drugs include improved solubility and pharmacokinetics,^105^ enhanced efficacy and lower drug doses, and reduced toxicity and increased selectivity. Several nanomedicines are currently on the market or in clinical trials.

Without a doubt, nanotechnology provides an accurate detection method as well as a new drug delivery system that is more targeted, efficient, and has fewer side effects. We anticipate that the use of nanotechnology‐based medicine will grow and that new nanomaterials will be developed, but that, as with other drugs and products, a more thorough risk assessment will be required before they are approved for clinical and commercial use.

## 3 TOXIC EFFECTS OF NPs ON HEALTH

With the discovery of CNTs, studies on the toxicity of NPs to human health began in the 2000s.^106^ Since then, researchers have conducted studies to assess the potential adverse effects of NPs exposure on various organs, including the lungs, liver, kidneys, and brain.^107^, ^108^ Studies have shown that NPs can enter the human body through inhalation, ingestion, and skin contact, causing damage to cells, tissues, and organs. Recent studies have focused on understanding the mechanisms of NPs toxicity and identifying factors that contribute to their adverse effects.^109^ One of the main findings is that the size, shape, surface area, and chemical composition of NPs are key determinants of their toxicity. In addition, the interactions of NPs with biological systems such as proteins, enzymes, and DNA can affect their toxicity. The next step in NPs toxicity research is to develop strategies to mitigate the risks associated with NPs exposure. This includes developing safe and efficient methods for NPs synthesis, identifying biomarkers of NPs toxicity, and developing protective measures for workers in industries that use NPs. In addition, more research is needed to understand the long‐term effects of NPs exposure on human health and the environment. In conclusion, NPs are a double‐edged sword, as they have the potential to revolutionize fields, but at the same time, they pose significant health risks. Therefore, it is critical to continue to study the toxic effects of NPs on human health and to develop strategies to mitigate these risks. This part discusses the influence of NPs exposure on the respiratory system, nervous system, endocrine system, immune system, and reproductive system, as well as the relationship with the occurrence and development of tumors. A multifaceted review of the effects of NPs exposure on human health has been conducted.

### 3.1 Toxic effects of NPs on the respiratory system

The respiratory system is a general term for a series of organs that exchange gas between the human body and the outside air,^110^ including the nose, pharynx, larynx, trachea, bronchi, and lungs composed of a large number of alveoli, blood vessels, lymphatic vessels, and nerves, as well as pleura and other tissues.^111^ The deposition of NPs in the respiratory system mainly depends on their size, shape, and surface chemistry. In general, smaller NPs are more likely to be deposited in the lower airways, where they can penetrate the alveolar region and interact with lung cells.^112^ The surface chemistry of NPs also affects their deposition, as particles with hydrophilic surfaces are less likely to be deposited in the respiratory system than hydrophobic particles. In addition, particle shape also affects their deposition, with elongated particles having a higher probability of deposition in the respiratory system compared with spherical particles. When NPs are deposited in the respiratory system, they can interact with different types of lung cells, such as alveolar macrophages, epithelial cells, and fibroblasts. These cells can recognize and engulf NPs through phagocytosis or pinocytosis.^113^ The uptake of NPs can trigger a series of cellular responses, such as the generation of ROS, the release of cytokines, and activation of inflammatory pathways. Cellular responses to NP exposure are highly dependent on particle size, surface chemistry, and dose. Exposure to NPs has been linked to various adverse effects on the respiratory system, such as inflammation, oxidative stress, fibrosis, and even lung cancer. Adverse effects of NP exposure depend largely on the physicochemical properties of the particles and the exposure dose. For example, titanium dioxide (TiO_2_)‐NPs have been shown to induce lung inflammation and fibrosis in animal studies.^114^ Adverse respiratory effects of NP exposure are also exacerbated by preexisting respiratory diseases, such as asthma^115^ or chronic obstructive pulmonary disease.^116^

In recent years, biomarkers have been used as a powerful tool to study the interaction between NPs and their related health outcomes.^117^ They are widely used to explore the interlinkages between environmental stimuli and adverse health events.^118^ Overall, the adverse effects of NPs on the respiratory system are mainly manifested in the following aspects: oxidative stress, inflammation, respiratory epithelial damage, fibrosis, and genetic changes.^119^ Previous studies have shown that NPs such as SiO_2_‐NPs,^120^ carbon‐based NPs,^121^ Ag‐NPs, and ZnO‐NPs^122^ can cause damage to the respiratory system, such as respiratory immune toxicity and inflammatory reactions. The study by Xu et al.^123^ showed that two kinds of nanoplastic particles with different diameters were internalized by human lung epithelial cells and induced cell cycle arrest and apoptosis. This is consistent with the study by Liu et al.^124^ that SiO_2_‐NPs exposure can penetrate the air–blood barrier in the lungs and enter the systemic circulation, thereby invading the cardiovascular system and producing cardiotoxic effects. Exposure to Ag‐NPs of different sizes can cause respiratory difficulties and increase the expression of the Hsp70 protein in Drosophila. Similarly, studies by Panacek et al.^125^ have shown that Ag‐NPs can cause serious adverse effects on the respiratory system of Drosophila. Several studies have shown that intratracheal instillation of CuO‐NPs can induce oxidative stress, inflammation, and tumor lesions in rats.^126^ NPs not only cause damage to the respiratory system but also disrupt the balance of the immune system, causing immunosuppression or overactivation of the immune system, thereby reducing the body's ability to resist respiratory viruses.^127^ In addition to NPs, other nanomaterials may also cause damage to the body by affecting the respiratory system, such as CNTs^128^ and so on. Numerous studies have demonstrated the role of CNTs in remodeling the respiratory system. CNT alone or in allergen‐sensitized animal models promotes airway and lung remodeling through the recruitment of cytokines tumor necrosis factor alpha (TNFα), interleukin 1 beta (IL1‐β), monocyte chemoattractant protein‐1, IL‐13, and blood inflammatory cells.^129^, ^130^, ^131^, ^132^, ^133^, ^134^ CNT‐induced extracellular remodeling is mainly mediated through transforming growth factor‐beta (TGFβ), TNFα, and osteopontin signaling pathways.^135^, ^136^ Studies by Halimu et al.^137^ have shown that nanoplastics in the air are easily inhaled and accumulate in the alveoli of humans and animals and induce the epithelial–mesenchymal transition of human alveolar epithelial A549 cells through NADPH oxidase 4 (NOX4) and ROS, thereby promoting the occurrence of pulmonary fibrosis. This is consistent with the study by Lin et al.^138^ that nanoplastics can induce mitochondrial dysfunction in lung cells and metabolic toxicity in target human cells. Compared with SiO_2_‐NPs‐exposed rats, TiO_2_‐NPs‐exposed rats exhibited significantly severe pulmonary alveolar proteinosis pathological changes, lower fibrosis, and higher levels of inflammatory biomarkers. However, SiO_2_‐NPs‐exposed rats had more severe fibrotic lesions and more severe granulomas than TiO_2_‐NPs‐exposed rats.^139^ In summary, NPs benefit from their physical properties and can easily enter the systemic circulation through the respiratory system through the air–blood barrier, causing serious adverse effects on the respiratory system. The adverse effects of NP exposure on the respiratory system are largely dependent on particle size, surface chemistry, and dose. It is worth mentioning that masks may be an effective way to prevent NPs with a diameter greater than 300 nm from entering the body through the respiratory tract. But there is no effective way to prevent smaller NPs from causing damage to the respiratory system.

### 3.2 Toxic effects of NPs on the nervous system

The nervous system—especially the brain and its cognitive abilities—is one of the most unique and impressive attributes of humans.^140^ It is the system that plays a leading role in the regulation of physiological functions and activities in the body. It is mainly composed of nervous tissue and is divided into two parts: the central nervous system and the peripheral nervous system. The central nervous system includes the brain and spinal cord, and the peripheral nervous system includes the cranial and spinal nerves.^141^ Neurological and psychiatric disorders are increasingly associated with a range of systemic comorbidities,^142^, ^143^ the most prominent of which are immunological^144^ and bioenergetic parameters^145^ as well as impairment of the gut microbiome.^146^ The research on the interaction between NPs and the nervous system mainly focuses on the application of NPs in drug delivery.^147^ Numerous studies have shown that NPs can cross the blood–brain barrier (BBB),^148^, ^149^ thereby entering the brain and affecting the nervous system.^147^, ^150^ Partial NPs can avoid phagocytosis by the reticuloendothelial system (RES) and significantly increase drug concentration in the brain.^151^ For example, modification with polyethylene glycol can prolong the retention time of liposomes in blood.^152^ Although the research on NPs in the direction of drug delivery is very hot, the effect of NPs on neurotoxicity cannot be ignored. The mechanisms of NPs‐induced neurotoxicity are diverse.^153^ Recent in vitro studies have shown changes in morphology, cell death, genotoxicity, oxidative stress, and proinflammatory responses after exposure to NPs.^154^, ^155^, ^156^ Teng et al.^157^ showed that polystyrene NPs (PS‐NPs) induced intestinal inflammation, growth inhibition, and developmental restriction in zebrafish, which were closely related to dysregulation within the brain–gut–microbiota axis. SiO_2_‐NPs entered the brain by intranasal instillation and accumulated in the striatum. Exposure to SiO_2_‐NPs also resulted in increased oxidative damage and striatal inflammatory response.^158^ Meanwhile, in vitro, results showed that exposure to SiO_2_‐NPs decreased cell viability, increased lactate dehydrogenase levels, triggered oxidative stress, disrupted the cell cycle, induced apoptosis, and activated p53‐mediated signaling pathways.^159^ Sobolewski et al.^160^ showed that exposure to Fe_2_O_3_‐NPs caused oxidative damage and neurotoxicity in the mouse brain. In addition to the above‐mentioned NPs, NPs such as Ag‐NPs,^161^, ^162^ PbO‐NPs,^163^ and ZnO‐NPs^164^ can cause damage to the nervous system to vary degrees. Taken together, NPs can be inhaled through the nose and mouth to cross the blood–brain barrier and cause neurological damage.

### 3.3 Toxic effects of NPs on the endocrine system

The endocrine system consists of multiple endocrine tissues, including not only traditional endocrine organs, but also discrete neuroendocrine cell lesions, including neuroendocrine tumors of the lung, gastrointestinal tract, thymus, breast, and prostate as well as paraganglia and adrenal glands.^165^, ^166^ These tissues produce and secrete hormones directly into the blood circulation to regulate bodily functions. The endocrine system cooperates with the nervous and immune systems^167^ to regulate different physiological processes such as maintaining homeostasis,^168^ regulating energy balance,^169^, ^170^ development, growth,^171^ and reproduction.^172^ NPs have effects on the endocrine system of mammals and other species, some of which are unfavorable or unwanted and others beneficial.^173^ NPs can interact with the endocrine system through multiple mechanisms. One such mechanism is their ability to mimic hormones. NPs can enter cells and bind to hormone receptors, thereby activating or inhibiting downstream signaling pathways. For example, some metal‐based NPs, such as Ag ZnO, have been shown to bind to estrogen receptors and exert estrogenic activity. This can disrupt the endocrine balance, especially in sensitive groups such as pregnant women and children. Another mechanism of action is the induction of oxidative stress. NPs can generate ROS in cells, leading to cell damage and disruption of signaling pathways. In addition, NPs interfere with the function of enzymes and transporters involved in endocrine regulation. For example, carbon‐based NPs have been shown to inhibit the activity of aromatase, an enzyme critical for estrogen biosynthesis. This can lead to lower estrogen levels and endocrine dysfunction. Lei et al.^174^ showed that TiO_2_‐NPs enhanced the thyroid endocrine‐disrupting effect of PCP exposure in zebrafish. Zhu et al.^175^ showed that SiO_2_‐NPs, even at nontoxic concentrations, increased the thyroid glands of juvenile zebrafish coexposed to PCBPA by promoting the bioaccumulation and bioavailability of PCBPA hormone disruption. Miao et al.^176^ showed that TiO_2_‐NPs increased the bioconcentration of lead, which led to the disruption of thyroid endocrine and neuronal systems in larval zebrafish. Studies have found that Ag‐NPs disrupt male reproductive endocrine balance through the hypothalamic–pituitary–gonadal axis^177^ and direct testicular cell damage.^178^, ^179^ Studies by Hussein et al.^180^ showed that ZnO‐NPs acted on Leydig cells to reduce steroidogenesis in mice under in vivo conditions.^181^ In contrast, ZnO‐NPs had the effect of enhancing steroidogenesis in TM‐3 cells in vitro.^182^ Taken together, disruption of NPs and endocrine function is associated with adverse health outcomes, including reproductive failure and metabolic syndrome. However, there are relatively few reports on the mechanism of NPs’ damage to the endocrine system. Therefore, further studies are needed to thoroughly clear any potential risk of various NPs for pathological endocrine disruption.

### 3.4 Toxic effects of NPs on the immune system

Immunity is divided into innate immune response and acquired immune response. Innate immunity refers to a nonselective rejection and clearance function of the body against antigenic substances entering the body, including immune system elements (neutrophils, monocytes, macrophages, complement, cytokines, and acute phase protein).^183^ The high conservation of this response, seen in even the simplest animals, confirms its importance for survival. Acquired immune response, also called specific immunity, is a sign of the immune system of higher animals. This response includes antigen‐specific responses by T lymphocytes and B lymphocytes.^184^, ^185^ Specific immunity is precise but takes days or weeks to develop. NPs can be recognized by immune cells to regulate the immune response,^186^ but this regulation is mostly reported in the innate immune response.^187^, ^188^, ^189^, ^190^ Auffret et al.^191^ showed that exposure to cadmium NPs (Cd‐NPs) resulted in decreased hemophagocytosis and immunosuppression in oysters (Crassostrea Gigas). This is consistent with the study by Bruneau et al.^192^ that Cd‐NPs caused a severe decrease in the viability of monocytes in mice, accompanied by lymphocyte transformation, resulting in immunodeficiency. Yamawaki et al.^193^ showed that after exposure to carbon black NPs, increased levels of proinflammatory cytokines (TNF‐α) and chemokines and decreased phagocytic capacity in macrophages were observed. Benmerzoug et al.^194^ showed that SiO2‐NPs‐induced sterile lung inflammation exacerbates M. tuberculosis infection through STING‐dependent type 2 immunity. In addition to the above NPs, NPs such as PS‐NPs, ZnO‐NPs,^195^ Ag‐NPs,^196^ and Au‐NPs^197^ can also activate innate immunity, including but not limited to promoting inflammatory factors, transcription, and translation of antimicrobial peptides, chemokines, and cytokines. Notably, Au‐NPs morphologies with the same surface chemistry elicited different innate immune responses in different environments in vivo and in vitro. This may be related to the complex interactions between Au‐NPs and other systems in vivo.^198^ In summary, most NPs can activate the innate immune response and induce the expression of inflammatory factors in cells, resulting in overactivation or immunosuppression of the innate immune response. However, there are few reports on the regulation of NPs on specific immunity.

### 3.5 Toxic effects of NPs on the reproductive system

Reproductive organs achieve the function of reproduction through various activities, fertilization, pregnancy, and other physiological processes. The function of the male reproductive system is mainly to produce sperm and transport sperm, while the function of the female reproductive system is mainly to ovulate and conceive.^199^ NPs can enter the circulatory system through various routes, and finally penetrate the Sertoli cell barrier to reach the reproductive system and cause toxic effects.^200^ There is growing evidence that exposure to NPs can negatively affect the reproductive system. The exact mechanism by which NPs affect the reproductive system is not fully understood. However, NPs can cause damage to DNA, which can lead to cell death or mutation. This can lead to infertility or other reproductive disorders. NPs can also cross the blood–testis barrier, which is designed to protect the testes from harmful substances.^201^ The presence of NPs in the testes induces inflammation and oxidative stress, which leads to cellular damage and dysfunction. This can lead to a drop in sperm count and motility, which can lead to male infertility. In women, NPs also affect the reproductive system. They can cross the placental barrier into the fetal blood and have long‐term effects on the reproductive system of the offspring.^202^ NPs can also cause damage to the ovaries, resulting in decreased egg quantity and quality. This can lead to infertility or other reproductive disorders. Due to their antimicrobial properties, Ag‐NPs are commonly used in consumer products such as clothing and cosmetics. However, exposure to Ag‐NPs has been shown to have negative effects on the reproductive system.^203^ Numerous studies have shown that NPs can cross biological barriers protecting reproductive tissues and accumulate in the testis,^204^ and cause oxidative stress, sex hormone disturbances, inflammation, and germ cell damage.^173^ For example, Au‐NPs,^205^ Ag‐NPs,^206^, ^207^ CNTs,^207^, ^208^ SiO_2_‐NPs,^209^ ZnO‐NPs,^210^ and cerium oxide NPs^211^ (CeO_2_‐NPs) could enter the testis, while Au‐NPs, Ag‐NPs, TiO_2_‐NPs, and CeO_2_‐NPs could continuously accumulate in the testis to cause toxicological damage. Among them, except CeO_2_‐NPs can induce spermatogenesis disorder in mice, the reproductive toxicity of other types of NPs is mainly due to tissue damage and inflammatory response. It is worth noting that any NP would need to cross the Sertoli cell barrier to enter the vas deferens, and this process may also involve stromal cells.^212^ Taken together, NP exposure‐induced reproductive toxicity is ubiquitous, which is related to the unique physicochemical properties of NPs, which can cross the Sertoli cell barrier and cause reproductive organ damage and persistent inflammatory responses. However, the prevention and control measures for the reproductive toxicity of NPs have not been reported yet. It is important to further study the effects of NPs on the reproductive system and develop appropriate safety measures to minimize the risks associated with their use.

### 3.6 Carcinogenicity of NPs

Cancer is a complex disease that affects millions of people worldwide and remains a leading cause of death despite significant advances in treatment and prevention. Carcinogenesis is the process by which normal cells turn into cancer cells. It is a complex process involving multiple steps, including initiation, ascension, and progression. Initiates genetic or epigenetic changes involving normal cells, leading to the development of precancerous lesions. Promotes the growth and enlargement of precancerous lesions involved, leading to the development of benign tumors. Progression involves the acquisition of additional genetic or epigenetic alterations that lead to the development of malignancy. Some NPs have also been shown to be carcinogenic in nature, and this damaging effect is due to the induction of damage at the DNA level by ROS, mutation, apoptosis, cell cycle inhibition, enhanced secretion of cytokines and chemokines, inflammatory responses, immunosuppression, and reduced viability of major cell types involved in the innate and adaptive immune system.^213^, ^214^, ^215^ It is worth noting that compared with other types of NPs, the reports on the carcinogenicity of NPs are mainly concentrated in metal NPs, which may be related to the chemical properties of the NPs themselves. For example, NPs themselves can also exhibit carcinogenicity at non‐nanometer sizes. The carcinogenicity of other types of NPs besides metal NPs such as ZnO‐NPs,^216^ hexavalent chromium NPs,^217^ and nickel NPs (Ni‐NPs)^218^ needs further research. And what needs to be paid attention to in future research is the relationship between the carcinogenicity of NPs and the properties of the substance itself and the size of the nanometer scale. The occurrence and development of cancer is a long process, which may be the reason why there are few reports on the carcinogenicity of NPs.

**FIGURE 1:**
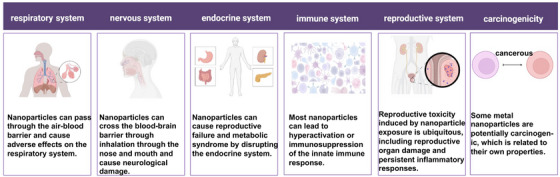
Toxic effects of nanoparticles on health.

In recent years, the toxic effects of NPs on human health have been extensively studied. NP has been found to cause a range of adverse effects, including respiratory disease, cardiovascular disease, neurological disease, and cancer (Table 1). The mechanisms underlying the toxic effects of NPs are complex and depend on various factors such as size, shape, surface chemistry, and physicochemical properties. The toxicological mechanism of NPs is still not fully understood. However, several studies have shown that the toxicity of NPs is mainly due to their ability to generate ROS and induce oxidative stress. ROS are highly reactive molecules that can damage cellular components such as proteins, lipids and DNA, leading to cell death or dysfunction. NPs can also induce inflammation by activating the immune system and releasing proinflammatory cytokines (Figure 1). In conclusion, the toxic effect of NPs on human health has attracted more and more attention, and its toxicological mechanism is complex and multifactorial. Further research is needed to understand the underlying mechanism and develop effective strategies to mitigate its adverse effects. The development of safe and biocompatible NPs is crucial for their successful application in various fields such as medicine, electronics, and energy.

**TABLE 1:** Effects of nanoparticles on various systems of the body.

Type of NPs	Size (nm)	Study design	Action object	Findings	References
SiO_2_‐NPs	220 nm	Animal experiment	Respiratory system	SiO_2_‐NPs specifically adsorb apolipoprotein AI (Apo AI) in blood to improve its cytotoxicity, while rapid clearance of SiNPs from blood depletes plasma Apo AI and promotes SiNPs‐induced atherosclerosis.	^124^
CuO‐NPs	50 nm	Animal experiment	Respiratory system	Intratracheal instillation of copper oxide nanoparticles induces oxidative stress, inflammation, and tumor lesions in rats.	^126^
PS‐NPs	100 nm	Animal experiment	Nervous system	Polystyrene nanoparticles induce intestinal inflammation, growth inhibition, and developmental restriction in zebrafish, which are closely related to dysregulation within the brain–gut–microbiota axis.	^157^
SiO_2_‐NPs	20 nm	Animal experiment	Nervous system	SiO_2_‐NPs entered the brain by intranasal instillation and accumulated in the striatum. Exposure to SiO_2_‐NPs also resulted in increased oxidative damage and striatal inflammatory response.	^158^
SiO_2_‐NPs	20 nm	Cell experiment	N/A	SiO_2_‐NPs decreased cell viability, increased lactate dehydrogenase levels, induced oxidative stress, disrupted the cell cycle, induced apoptosis, and activated p53‐mediated signaling pathways.	^159^
Fe_2_O_3_‐NPs	150 nm	Animal experiment	Nervous system	Iron oxide nanoparticles exposure can cause oxidative damage and neurotoxicity in the mouse brain.	^160^
SiO_2_‐NPs	20 nm	Animal experiment	Endocrine system	SiO_2_‐NPs increase thyroid hormone disruption in juvenile zebrafish coexposed to PCBPA by promoting PCBPA bioaccumulation and bioavailability.	^175^
TiO_2_‐NPs	150 nm	Animal experiment and cell experiment	Endocrine system and nervous system	TiO_2_‐NPs increased the bioconcentration of lead, which led to the disruption of thyroid endocrine and neuronal systems in larval zebrafish.	^176^
Cd‐NPs	N/A	Animal experiment and cell experiment	Immune system	Cd‐NPs exposure resulted in decreased hemocyte phagocytosis in Crassostrea gigas and resulted in immunosuppression.	^191^
Cd‐NPs	N/A	Cell experiment	Immune system	Cd‐NPs lead to a severe decrease in the viability of monocytes in mice, accompanied by lymphocyte transformation, resulting in immunodeficiency.	^192^
carbon black nanoparticles	170‐410 nm	Cell experiment	Immune system	After exposure to carbon black nanoparticles, increased levels of proinflammatory cytokines (TNF‐α) and chemokines and decreased phagocytic capacity were observed in macrophages.	^193^
NPs	N/A	N/A	Immune system	NPs can cross biological barriers that protect reproductive tissues and accumulate in the testes, causing oxidative stress, sex hormone disturbances, inflammation, and germ cell damage.	^204^
Au‐NPs	5 nm	Animal experiment	Immune system	Au‐NPs were able to internalize into endosomes/lysosomes of TM3 Leydig cells, induce autophagosome formation, increase reactive oxygen species (ROS) production, and disrupt the cell cycle in the S phase, resulting in condensation‐dependent cellular Toxicity and DNA damage. Moreover, AuNPs significantly decreased testosterone production in TM3 cells by inhibiting the expression of 17α‐hydroxylase, an important enzyme in androgen synthesis.	^205^

## 4 TOXICOLOGICAL MECHANISMS OF NPs

Research on the toxicological mechanism of NPs began in the early 2000s.^219^ The development of new analytical techniques, such as transmission electron microscopy and atomic force microscopy, has enabled researchers to study the interactions between NPs and cells in greater detail. Studies have shown that NPs can cause oxidative stress, inflammation, genotoxicity, and cytotoxicity in various cell types.^220^ Recent studies have focused on understanding the molecular mechanisms of NP toxicity. One of the main findings is that the surface chemistry of NPs plays a crucial role in their toxicity. Surface modification, such as coating with biocompatible materials, can reduce the toxicity of NPs. Furthermore, the formation of protein coronas around NPs in biological fluids also affects their toxicity. The toxicity of NPs can be roughly divided into acute toxicity and chronic toxicity. Acute toxicity refers to adverse effects such as inflammation and tissue damage immediately after exposure to NP. Chronic toxicity refers to the long‐term effects of exposure to NP, such as cancer and organ damage.^221^ The major types of toxicity caused by NPs include oxidative stress, inflammation, genotoxicity, and cytotoxicity. The next step in the study of the toxicological mechanism of NPs is to develop predictive models that can accurately predict the toxicity of NPs based on their physicochemical properties. This includes the development of in vitro models that can mimic the in vivo environment, and the use of high‐throughput screening (HTS) methods to rapidly identify the toxicity of large numbers of NPs. In addition, more research is needed to understand the long‐term effects of chronic NP exposure on human health. In conclusion, the toxicological mechanisms of NPs are complex and depend on multiple factors, such as their physicochemical properties and biological environment. The development of predictive and in vitro models that can accurately predict NP toxicity is critical for the safe and effective use of NP in a variety of applications.

### 4.1 Studies between NPs and ROS

ROS is a general term describing the chemical species formed by the incomplete reduction of oxygen, including superoxide anion (O^2−^), hydrogen peroxide (H_2_O_2_), and hydroxyl radical (HO).^222^ ROS can be produced in mitochondria, peroxisomes, endoplasmic reticulum (ER), and cytoplasm. In mitochondria, ROS are produced as a by‐product of electron transport during oxidative phosphorylation. In the peroxisome, ROS are generated during fatty acid metabolism, and in the ER, ROS are generated during protein folding. ROS are thought to mediate the toxicity of oxygen because of their greater chemical reactivity relative to oxygen. In vivo, ROS plays an important role in regulating cell signaling and cellular physiological functions.^223^ Excessive accumulation of ROS often destroys the oxidative‐antioxidant system of cells, leading to the occurrence of oxidative stress.^224^ Major cellular antioxidant defense systems include enzymes such as superoxide dismutase, catalase, and glutathione peroxidase. These enzymes help neutralize ROS and prevent them from causing damage. Oxidative stress can lead to cellular damage and dysfunction, leading to a variety of diseases. For example, in cardiovascular disease, oxidative stress leads to the oxidation of low‐density lipoprotein cholesterol, which promotes the development of atherosclerosis. In neurodegenerative diseases such as Alzheimer's disease, oxidative stress can lead to neuronal damage and cell death. ROS can play a dual role in cancer, acting as both tumor promoters and tumor suppressors. On the one hand, ROS can promote carcinogenesis by causing DNA damage, which leads to mutations and the activation of oncogenes. On the other hand, ROS can suppress cancer by inducing cell death and preventing cancer cell proliferation.

In many cases, cancer cells exhibit higher levels of ROS than normal cells, which makes them more susceptible to oxidative stress. Therefore, targeting ROS production or increasing antioxidant defense systems in cancer cells has been proposed as a potential therapeutic strategy for cancer. ROS are implicated in the aging process, and excessive ROS production can lead to cellular damage and dysfunction, leading to age‐related diseases. However, ROS also plays a role in normal aging, acting as signaling molecules that regulate cellular processes. Caloric restriction has been shown to extend lifespan in various animal models, and it is thought to reduce ROS production and increase antioxidant enzyme activity. This suggests that reducing ROS production and increasing antioxidant defenses may be potential strategies to promote healthy aging. More and more studies have shown that exposure to NPs causes ROS accumulation.^225^, ^226^, ^227^ The study by Liu et al.^228^ showed that silica NPs (SNPs) induced blood–brain barrier dysfunction in vitro and in vivo and produced massive ROS accumulation causing oxidative stress. Yang et al.^229^ showed that coexposure to PS–NPs damaged the fetal thalamus by inducing ROS‐mediated apoptosis. It has also been shown that ZnO‐NPs^230^ and CuO‐NPs^231^ can cause hepatotoxicity and induce ROS accumulation and oxidative stress. In addition to the above substances, various NPs such as carbon NPs,^232^ titania NPs,^233^, ^234^ nickel oxide NPs,^235^ and CeO_2_‐NPs^236^ can cause the accumulation of ROS and induce Oxidative stress. As research reports gradually increased, scientists found that not all NPs played a positive role in the accumulation of ROS. Studies have shown that polydopamine NPs can act as efficient scavengers of ROS in periodontal disease,^237^ and cyclic polysaccharide β‐cyclodextrin NPs reduced systemic and local oxidative stress and inflammation, as well as reduced atherosclerosis Inflammatory cell infiltration in plaques.^238^ The common feature of these NPs with antioxidants effect is that these substances themselves have an antioxidant effect. When substances with oxidative effects exist in the state of NPs, their antioxidant effects will be amplified because the NPs are easily internalized by cells, which is one of the reasons why nanomaterials are used in medicine.^225^, ^239^ The above results show that the regulation of ROS by different types of NPs is different, which is related to the properties of the substances themselves. The effects of the same NPs of different sizes on ROS are also different, and the representative substances are nanoplastics.^240^ Taken together, exposure to NPs formed by nonantioxidant substances can induce intracellular ROS accumulation, disrupt the balance of the oxidative‐antioxidant system, and cause oxidative stress. NPs formed by antioxidant drugs maximize their antioxidant effects by the easy internalization of NPs by cells.

### 4.2 Studies between NPs and mitochondrial damage

Mitochondria are important organelles of eukaryotic cells and are the key to ATP production, and their activities are strictly controlled.^241^ Mitochondrial damage can be caused by a variety of causes, including genetic mutations, oxidative stress, impaired mitochondrial dynamics, and toxic insults. Genetic mutations in nuclear or mitochondrial DNA can lead to defects in mitochondrial proteins, lipids, or nucleic acids, compromising mitochondrial integrity and function. Oxidative stress is caused by an imbalance between the production of ROS and the cellular antioxidant defense system, which can cause damage to mitochondrial membranes, proteins, and DNA. Impaired mitochondrial dynamics, which refers to the balance between mitochondrial fusion and fission, may also lead to mitochondrial damage by altering mitochondrial morphology and function. Exposure to toxic substances such as drugs, chemicals, or environmental pollutants can directly damage mitochondrial components, leading to mitochondrial dysfunction.

Mitochondrial dysfunction can have profound effects on cellular physiology, as mitochondria play key roles in energy metabolism, calcium signaling, and apoptosis. Reduced ATP production and impaired oxidative phosphorylation can lead to decreased cellular energy levels and contribute to the development of metabolic disorders such as diabetes and obesity. Dysregulation of calcium signaling, which is tightly regulated by mitochondria, disrupts cellular homeostasis and contributes to the development of neurodegenerative diseases such as Alzheimer's and Parkinson's. Mitochondrial dysfunction also leads to dysregulation of apoptosis, a key mechanism of programmed cell death. Dysregulation of apoptosis is associated with a variety of diseases, including cancer, cardiovascular disease, and neurodegenerative diseases. These include mitochondrial proteases, proteasome‐mediated outer mitochondrial membrane protein degradation, mitochondrial‐derived vesicle degradation, and mitophagy.^242^ More and more studies have found that NPs can affect mitochondrial function or cause mitochondrial damage to cause apoptosis.^243^ Recent studies have shown that nanocomposites disrupt the Ca+ buffering function of mitochondria in tumor‐associated macrophages, triggering calcium overload and causing mitochondrial damage. Huang et al.^244^ showed that decabromodiphenylethane and ZnO‐NPs reduced mitochondrial membrane potential (MMP), increased cytochrome C release, and modulated Bax/Bcl‐2 and cysteine by disrupting mitochondrial kinetic homeostasis Apoptosis was induced by the expression of dp‐3 mRNA and protein. Fu et al.^245^ showed that amino‐functionalized PS–NPs induced mitochondrial damage and decreased cell viability in human umbilical vein endothelial cells. This is consistent with the study by Li et al.^246^ that PS microplastics trigger mitochondrial damage and apoptosis through ROS‐driven calcium overload. Wu et al.^247^ showed that Fe_3_O_4_‐NPs can cause mitochondrial damage and reduce macrophage viability after 48 h treatment, inducing a shift of macrophage polarization to the M1 phenotype. It has been reported that SiO_2_‐NPs can induce MMP depolarization in cardiomyocytes and reduce ATP production, resulting in Ca^2+^ damage.^248^ Li et al.^246^ showed that nanosized carbon black, zinc dioxide, and silica can all cause ROS accumulation and mitochondrial damage in human corneal epithelial cells and human conjunctival epithelial cells to vary degrees, but TiO_2_‐NPs have no toxic effect. This is consistent with the previous discussion that the toxicity of NPs depends on the properties of the substance itself, including physical and chemical properties. Therefore, not all nanosized substances cause bad effects. Although existing research can demonstrate that many NPs can cause mitochondrial damage. But how NPs work in this process has not yet been reported. For example, whether NPs can attach to mitochondria and alter their membrane surface structure to affect mitochondrial function. To sum up, to study the damage of NPs to mitochondria, it is not only necessary to study the damage caused by the NPs themselves but also to explore the damage of the non‐nano form of the same substance to the same target, from the perspectives of physical properties and chemical properties. A dialectical analysis of the mechanisms by which NPs cause damage.

### 4.3 Studies between NPs and inflammation

Inflammation is an adaptive response triggered by harmful stimuli and conditions, such as infection and tissue damage.^249^ This process often results in recovery and healing from infection, however, if targeted destruction and adjuvant repair are not properly staged, inflammation can lead to persistent tissue damage by white blood cells, lymphocytes, or collagen.^250^ Inflammation is closely related to immunity and is critical to maintaining the body's health. The inflammatory response is a complex series of events that occurs in response to tissue injury, infection, or other stimuli. The immune system plays a key role in initiating and modulating inflammatory responses, and in addressing inflammation after the threat has been eliminated. Inflammatory injury and immunity are often discussed in the context of disease because both are important factors in the pathogenesis of many diseases. Chronic inflammatory diseases, such as rheumatoid arthritis, inflammatory bowel disease, and asthma, are characterized by persistent inflammation that leads to tissue damage and dysfunction. In these diseases, the immune system is dysregulated and normal mechanisms of inflammation and immune function are disrupted. The inflammatory response is triggered by the recognition of danger signals, such as pathogen‐associated molecular patterns or damage‐associated molecular patterns, by pattern recognition receptors on immune cells. This recognition leads to the activation of signaling pathways that lead to the production of proinflammatory cytokines, chemokines, and other inflammatory mediators. Immune cells, including macrophages, dendritic cells, and T cells, play a key role in the initiation and regulation of inflammatory responses. Macrophages are responsible for phagocytosis of pathogens and the production of proinflammatory cytokines, while dendritic cells are important for antigen presentation and T cell activation. T cells participate in the adaptive immune response and can differentiate into various subsets with different functions, including regulatory T cells (Treg) that suppress inflammation and effector T cells that promote inflammation. In addition to immune cells, other cell types, including endothelial cells and fibroblasts, also contribute to inflammatory responses by producing cytokines, chemokines, and adhesion molecules that recruit immune cells to sites of injury or infection. In chronic inflammatory diseases, the normal mechanisms of inflammation and immune function are disrupted, resulting in persistent inflammation and tissue damage. The underlying causes of these diseases are complex and multifactorial, but dysfunctional immune cells and production of proinflammatory cytokines are key factors. In rheumatoid arthritis, for example, immune cells are activated by unknown triggers, leading to the production of proinflammatory cytokines such as TNF‐α and IL‐1. These cytokines promote joint inflammation and tissue destruction, leading to pain, swelling, and eventually joint deformity. In inflammatory bowel disease, intestinal epithelial dysfunction leads to the activation of intestinal immune cells, which produce proinflammatory cytokines that damage the intestinal lining. This can lead to chronic inflammation and symptoms such as diarrhea, abdominal pain, and weight loss. It has been reported that NPs may induce inflammatory effects through immune cells.^189^ In this process, macrophages are the main responders of NPs.^251^ Because of this, more and more scholars take macrophage‐targeted nanomedicine as a research direction for the treatment of diseases.^252^, ^253^, ^254^ However, NPs that people usually come into contact with in the environment can induce inflammation in cells or individuals, such as PS NPs,^254^ SNPs,^255^ and carbon NPs.^121^ The study by Wu et al.^256^ showed that PS‐NPs with different diameters can act on the TLR4/NOX2 signaling pathway to induce oxidative stress, and further trigger the Th1/Th2 imbalance in carp myocardial tissue, resulting in inflammatory damage. This is consistent with Tang et al.’s study^257^ that PS nanoplastics exacerbate lipopolysaccharide‐induced spleen necrosis and inflammation in mice via the ROS/MAPK pathway. It has been reported that the main toxicities of nano‐TiO are genotoxicity, membrane damage, inflammation, and oxidative stress.^258^ In addition, Zhang et al.^259^ found that high‐concentration (100 μg/ml) graphene oxide exposure resulted in intraocular inflammation, corneal apoptosis, iris neovascularization, and corneal epidermal cell apoptosis in mice. Studies have shown that NPs can indirectly cause developmental toxicity through inflammation and oxidation.^260^ Although a large number of studies have shown that NPs can cause inflammatory damage, there are few reports on how NPs regulate inflammation‐related pathways.

### 4.4 Studies between NPs and apoptosis

Apoptosis, a form of programmed cell death, represents a key tumor suppressor mechanism that is activated in response to stress signals (e.g. DNA damage, ER stress),^261^ essential for the normal development and function of multicellular organisms.^262^ With the gradual deepening of NP research, more and more studies have shown that NP exposure can induce apoptosis.^263^, ^264^, ^265^, ^266^ The study by Xu et al.^267^ showed that ZnO‐NPs could induce ER stress in mouse ovarian cells through the Keap/Nrf2 signaling pathway and cause apoptosis. Yuan et al.^268^ showed that biosynthetic Ag‐NPs inhibited the malignant behavior of gastric cancer cells and enhanced the therapeutic effect of 5‐fluorouracil by promoting intracellular ROS generation and apoptosis. Li et al.^269^ showed that titania‐NPs enhanced testicular apoptosis and DNA damage caused by cypermethrin and promoted oxidative stress in testicular tissue. The latest study showed that the nano mix increased apoptosis and cell death, IL‐6, IL‐8, and TNFα expression, oxidative stress, and mitochondrial dysfunction compared with Ag‐NPs and PS‐NPs treatment alone, indicating that the mixture is an additive effect. Notably, the anti‐inflammatory cytokines IL1‐β, IL‐4, and IL‐10 were not affected by combined exposure compared with single NPs and Ag‐NPs could translocate into the nucleus causing strand DNA breaks.^270^ Zhang et al.^271^ found that SNPs can activate LC3 and Bax signaling pathways and cause apoptosis in RAW264.7 cells, while melatonin can promote autophagy and inhibit apoptosis caused by SNPs. This is consistent with the study by Ahamed et al.^272^ that coexposure to SNPs and arsenic can induce mitochondria‐dependent apoptotic toxicity. The study by Li et al.^273^ showed that exposure to nanoplastics decreased the intracellular ion content and the activity of ion‐transporting ATPase in Japanese giant short gill cells, and promoted the occurrence of apoptosis. Although the above studies have shown that NPs play a positive role in the occurrence and development of apoptosis, the specific molecular mechanism has not been reported. This may be related to the inability to unify the physical properties of NPs such as size and shape.

### 4.5 Studies between NPs and DNA damage

DNA damage, a phenomenon in which the nucleotide sequence of DNA is permanently altered during replication and results in altered genetic characteristics, has become a major culprit in cancer and many aging‐related diseases.^274^ Furthermore, DNA damage and other stresses can trigger a highly conserved, anticancer, antiaging survival response that inhibits metabolism and growth, enhances defenses, and maintains cellular integrity.^275^ A growing number of studies have shown that NP exposure causes DNA damage across cellular barriers.^276^, ^277^, ^278^ For example, TiO_2_‐NPs have only mild cytotoxic potential, but they can induce ROS and oxidative stress, leading to oxidative DNA damage.^279^ This is consistent with previous studies that TiO_2_‐NPs can induce oxidative DNA damage even at a concentration of 1 μg/mL. This effect can be attributed to decreased GSH levels and increased lipid peroxidation and ROS generation.^280^ One study showed that ultra‐small superparamagnetic iron oxide NPs can cause an imbalance of the oxidative‐antioxidant system in the mouse heart, increasing markers of oxidative stress and inducing DNA damage in the heart.^281^ Shi et al.^282^ showed that TiO_2_‐NPs can induce oxidative DNA damage in HepG2 cells and mice by inhibiting the Nrf2 pathway. This is consistent with previous studies that titania NPs induced DNA damage and genetic instability in mice.^283^ Mo et al.^284^ showed that Ni‐NPs exposure both in vitro and in vivo was involved in DNA damage and DNA repair through the HIF‐1α/miR‐210/Rad52 pathway, which was reflected in the expression of DNA damage response‐related proteins such as ataxia‐telangiectasia mutated proteins, p53, and H2AX. Phosphorylation increases. Ni‐NPs exposure also induced nuclear accumulation of HIF‐1α, upregulation of miR‐210, and downregulation of the homologous recombination repair gene Rad52. He et al.^285^ showed that CuO‐NPs induced oxidative DNA damage and cell death in HUVECs through copper ion‐mediated p38 MAPK activation. In addition, many NPs can induce DNA damage, such as Au‐NPs,^286^ hafnium oxide NPs,^287^ Ag‐NPs,^288^ SNPs,^289^ indium NPs,^290^ PS NPs,^291^ and so on. Notably, most NPs cause oxidative DNA damage by inducing oxidative stress.

### 4.6 Studies between NPs and the cell cycle

The cell cycle is a highly regulated process that controls the growth and division of cells in living organisms. This process is critical for the development, maintenance, and repair of tissues and the transmission of genetic material from one generation to the next. Defects in cell cycle control mechanisms lead to abnormal cell proliferation and may contribute to the development and progression of cancer and other diseases.

The cell cycle is divided into two main phases: interphase and mitosis. Interphase is the longest phase and is further divided into three subphases: G1, S, and G2. During the G1 phase, cells prepare for DNA replication and cell growth. During the S phase, DNA replication occurs and the cell synthesizes new proteins and organelles. During the G2 phase, cells prepare for cell division by synthesizing additional proteins and organelles.

Mitosis is the process by which a cell divides into two daughter cells, each containing an identical set of chromosomes. Mitosis is divided into four main phases: prophase, metaphase, anaphase, and telophase. During prophase, chromatin condenses into visible chromosomes and the nuclear envelope disintegrates. During metaphase, the chromosomes line up in the center of the cell on the metaphase plate. During anaphase, sister chromatids separate and are pulled to opposite poles of the cell. Finally, at telophase, the nuclear envelope around the daughter chromosomes reforms, and the cell undergoes cytokinesis to form two separate daughter cells.

The cell cycle is tightly regulated by multiple cellular signaling pathways, checkpoints, and feedback mechanisms. These regulatory mechanisms ensure that the cell cycle proceeds in a timely and orderly manner and prevent errors that could lead to DNA damage or abnormal cellular accumulation. At various points throughout the cell cycle, regulatory proteins and enzymes control the activity of key checkpoints that monitor DNA integrity, chromosome alignment, and cell size.

Many important proteins and enzymes are involved in regulating the cell cycle, including cyclins, cyclin‐dependent kinases (CDKs), and checkpoint kinases. Cyclins are a family of proteins that bind to CDKs to activate them and drive progression through the cell cycle. In turn, CDKs phosphorylate a variety of downstream targets, including proteins involved in DNA replication, chromosome segregation, and cell division. Checkpoint kinases monitor cell cycle errors and initiate DNA repair mechanisms or cell death pathways when necessary.

Cell cycle checkpoints function as DNA surveillance mechanisms that prevent the accumulation and spread of genetic errors during cell division. Checkpoints can delay cell cycle progression or induce cell cycle exit or cell death in response to irreparable DNA damage.^292^ PS‐NH2 NPs can cause cell cycle damage and joint arrest between G1/S and G2/M phases, and this arrest occurs gradually. Notably, despite the arrest of the cell cycle, intracellular ATP levels did not decline, nor did the internalization of NPs.^293^ The study by Holmila et al.^294^ showed that exposure to Ag‐NPs resulted in the accumulation of ROS, cell cycle arrest, and decreased cell proliferation in A549, BEAS‐2B, and Calu‐1, but not in NCI‐H358. This is consistent with the report by Lee et al.^295^ that Ag‐NPs induce ROS‐mediated cell cycle delay in *Candida albicans*. The study by Guo et al.^296^ showed that exposure to Si‐NPs leads to cell cycle arrest and apoptosis through the downregulation of cell cycle positive regulator expression and activation of TNF‐α/TNFR I.‐mediated apoptotic pathways. Palladium NP exposure caused a significant increase in cells in the G0/G1 phase and a significant decrease in the GS and G2/M phases of peripheral blood mononuclear cells.^297^ Moghaddam's study showed that ZnO‐NPs induced cell cycle arrest and apoptosis in the MCF‐7 cancer cell line.^298^ Recent studies have shown that carbon black NP exposure affects the cell cycle through circulating inflammation, thereby increasing lung cancer risk.^299^ The effect of NPs on the cell cycle may be related to the charge on their surface, and positively charged NPs are more likely to affect cell cycle progression by causing DNA damage.^300^ In summary, NPs can induce cell cycle arrest by inducing intracellular ROS accumulation and DNA damage, but the level of intracellular ATP may not decrease. There is a conjecture here that cells may cross the G2/M phase checkpoint after being exposed to NPs again, and cells continue to divide without DNA damage repair, but the relevant mechanism has not been reported.

### 4.7 Studies between NPs and epigenetic regulation

Epigenetic regulation refers to chemical modifications of DNA and histones that can affect gene expression without changing the DNA sequence itself. Epigenetic regulation is the process of regulating the content and function of intracellular nucleic acid or protein through epigenetic modification (such as methylation, acetylation, phosphorylation, etc.).^301^, ^302^ NPs can affect cellular function and activity through epigenetic regulation.^303^, ^304^ The study by Wu et al.^305^ showed that Au‐NPs induced epigenetic regulation of the PROS1 gene in lung fibroblasts, but the methylation status of this gene was unchanged in Au‐NPs‐treated fibroblasts. Pan et al.^306^ showed that Ag‐NPs were toxic to thermophiles through lipid peroxidation and mitochondrial dysfunction, identified 1250 differentially expressed lncRNAs in the process, and found that these lncRNAs exhibited toxicant‐specific expression mode. One of the best‐studied epigenetic modifications is DNA methylation, which involves the addition of methyl groups to cytosine nucleotides in DNA. DNA methylation can silence gene expression by preventing transcriptional machinery from accessing DNA. Studies have shown that exposure to certain NPs can alter DNA methylation patterns, which can lead to changes in gene expression. The study by Wang et al.^307^ showed that MET‐2, a methyltransferase, mediated methylation modulates the protective response to PS‐NPs exposure. MET‐2 functions to control the toxicity of PS‐NPs in both intestinal cells and germ cells. In intestinal cells, a MET‐2‐DAF‐16/BAR‐1/ELT‐2 signaling cascade is formed to control the toxicity of PS‐NPs. In germ cells, the MET‐2‐WRT‐3/PAT‐12 signaling cascade is necessary to control the toxicity of PS‐NPs.^307^ Wei et al.^308^ showed that CuO‐NPs exposure affected transgenerational developmental and reproductive toxicity in animals through epigenetically related genes met‐2 and spr‐5. In summary, NPs can regulate the life activities of cells or individuals through epigenetics, but the mechanism of NP regulation of epigenetics has not yet been reported. It is worth noting that it remains to be confirmed whether the epigenetic regulation is the same for different types of NPs.

**FIGURE 2:**
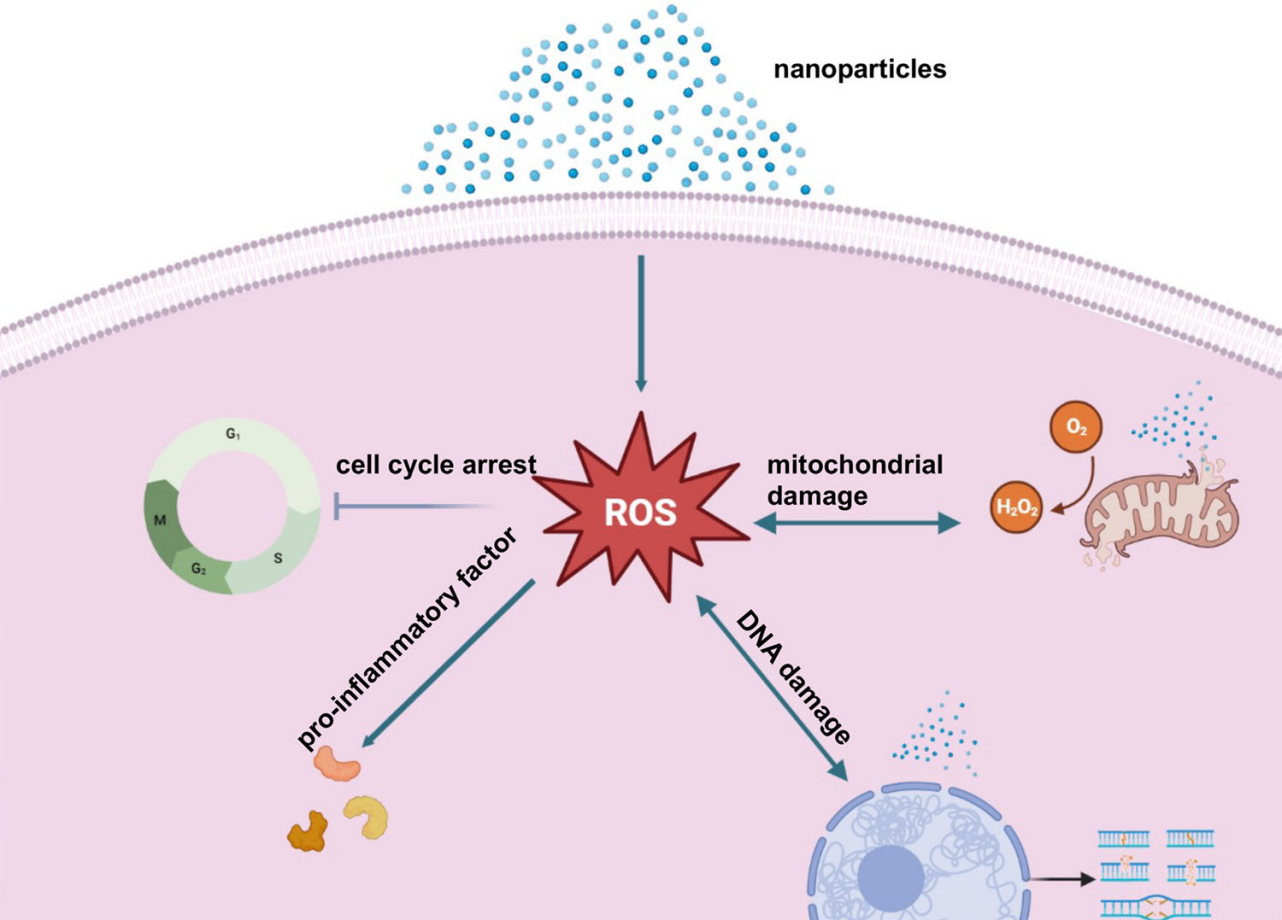
Nanoparticles are mainly related to the mechanism of toxicity induced by the accumulation of ROS.

The toxicological mechanism of NPs is inseparable from the physicochemical properties of the NPs themselves, and it is crucial to understand their potential adverse effects on human health. The above content summarizes the underlying mechanism of NP toxicity (Table 2), it is worth noting that NPs‐induced toxicity is often associated with the accumulation of ROS (Figure 2). Although current studies have made significant progress in understanding the toxicological mechanisms of NPs, effective toxicity assessment models are still needed to predict and assess potential adverse effects of NPs on human health. Common toxicity assessment models, such as animal models and in vitro models, have limitations in relation to human exposure and ethical concerns. To address these issues, researchers have proposed several NPs toxicity assessment models, including pharmacokinetic (PBPK) modeling, quantitative structure–activity relationship (QSAR) modeling, HTS analysis, and so on. Toxicity assessment models for NPs must also take into account uncertainties in human exposure scenarios, as well as interactions between NPs and organisms. The development of robust NPs toxicity assessment models requires interdisciplinary collaboration. In conclusion, understanding the toxicological mechanism of NPs is crucial for the safe and effective application of NPs. At the same time, developing a reliable and predictive NPs toxicity assessment model is also crucial for the study of its toxicity mechanism. The continuous development and improvement of these assessment models, as well as the integration of new technologies and methods, will enable a more comprehensive and accurate assessment of the potential adverse effects of NPs on human health and promote safer use of NPs.

**TABLE 2:** Toxicological effects of different types of nanoparticles on cells or individuals.

Type of NPs	Size (nm)	Concentration	Treatment time	Damage caused	In vitro/vivo	Mainly used experimental methods	Findings	References
SiO_2_‐NPs	20 nm	100 mg/mL	24 h	ROS, oxidative stress	Rat/HBMECs cells	Filamentous (F)‐actin staining and Western blot analysis	SiO_2_‐NPs can perturb the structure and function of the blood–brain barrier (BBB), induce BBB inflammation, and suggest that these effects may occur through ROS and ROCK‐mediated pathways.	^228^
SiO_2_‐NPs	100 nm	1 mg/day	17 days	ROS, oxidative stress, and apoptosis	C57BL/6 mice	Western blot analysis, Histopathological analysis, and immunohistochemistry/immunofluorescence	SiO_2_‐NPs lead to BBB paramembrane opening, oxidative stress, and activation of astrocytes in the brain. However, no obvious adverse effects on BBB structure and function were observed in the SiO_2_ MPs group.	^309^
ZnO‐NPs	100 nm	200 mg/kg/day and 400 mg/kg/day	90 days	ER stress and oxidative stress	C57BL/6 mice	Inductively coupled plasma mass spectrometry (ICP‐MS), ELISA, and Western blot analysis	Exposure to ZnO‐NPs resulted in increased levels of ER stress‐associated apoptotic proteins, such as caspase‐3, caspase‐9, caspase‐12, JNK phosphorylation, and CHOP/GADD153, and upregulated proapoptotic genes (chop and bax).	^230^
CuO‐NPs	357.42 ± 8.21 nm	10 μg Nano‐CuO/g body weight	60 days	Oxidative stress, endoplasmic reticulum stress, and apoptosis.	Male Wistar rats and BRL‐3A cells	ELISA and Western blot analysis	Cuo‐NPs treatment induced oxidative stress via ROS, which in turn led to the activation of the ER stress pathway, resulting in the death of liver tissue and BRL‐3A cells.	^231^
TiO_2_‐NPs	20 nm	50 μg/mL and 100 μg/mL	7 days	Oxidative stress, apoptosis, and cell cycle G2/M phase arrest	BALB/c mice	ROS detection, measurement of mitochondrial membrane potential, ELISA, cell cycle analysis, qPCR, and Western blot analysis	Critical role of Nrf2/ARE signaling pathway and functional proteins ZO‐1, Na‐K‐ATPase, and β‐catenin in TiO 2 NPs‐induced corneal endothelial cell injury.	^234^
PS‐NPs and NH_2_‐PS‐NPs	50 nm	20 μg/mL	24 h	Oxidative stress and mitochondrial damage	HUVEC cells	Mitochondrial membrane potential detection, ATP activity measurement, qPCR, and Western blot analysis	NH_2_‐PS‐NPs presented a higher risk to endothelial cells than uncharged nanoplastics by interfering with mitochondria.	^245^
PS‐NPs	5 μm	0.2, 0.4, and 0.6 mg/mL/0.5 mg/day	24 h/30 days	Ca^2+^ overload, oxidative stress, and apoptosis	L02 hepatocytes and C57BL/6 male mice	qPCR and Western blot analysis	Microplastics trigger hepatotoxicity by linking ROS to AMPK and Ca^2+^ signaling.	^246^
Fe_3_O_4_‐NPs	100 nm	200, 300, 400, and 500 μg/mL	24 h	Ferroptosis	Ana‐1 and LLC cell lines	ROS detection, qPCR, and Western blot analysis	p53 may contribute to Fe_3_O_4_‐NPs‐induced macrophage ferroptosis.	^247^
PS‐NPs	50, 100, and 400 nm	1000 μg/L	28 days	Oxidative stress, inflammation, and apoptosis	Cyprinus carpio	qPCR and Western blot analysis	PS‐NPs exposure can lead to oxidative stress, leading to carp cardiac inflammation and apoptosis, and the degree of damage is negatively correlated with the particle size of PS‐NPs.	^256^
PS‐NPs	100 nm	100 μg/mL	24 h	Cellular necrosis and inflammation	RAW264.7	qPCR and Western blot analysis	PS‐NPs promote LPS‐induced spleen necrosis and inflammation in mice via the ROS/MAPK pathway.	^257^
Ag‐NPs	23 ± 14 nm	10 μg/mL	24 h	Cell cycle arrest and mitochondrial damage	BEAS‐2B, A549, Calu‐1 and NCI‐H358	Mitochondrial respirometry analysis, cell proliferation, ATP assay, flow cytometry, and Western blot analysis	Exposure to Ag‐NPs resulted in cell cycle arrest and decreased cell proliferation in A549, BEAS‐2B, and Calu‐1, but not in NCI‐H358.	^294^
Si‐NPs	5−20 nm	10 mg/kg	7 days	Reproductive toxicity, oxidative stress, cell cycle arrest, and apoptosis	C57BL/6 mice	ROS measurement, flow cytometry analysis, qPCR, and Western blot analysis	Si‐NPs can cause testicular damage by inducing oxidative stress and DNA damage, resulting in cell cycle arrest and apoptosis, resulting in spermatogenic dysfunction.	^296^
Pd‐NPs	2−8 nm	0.1, 1, 5, 10, 20, 40, 80 μg/mL	48 h	Cell cycle arrest	Human peripheral blood mononuclear cells	ROS measurement and Cell cycle detection.	Low doses of Pd ions and Pd‐NP have potentially deleterious effects when hematopoietic cells leave quiescence.	^297^
ZnO‐NPs	100 nm	21 μg/mL	24 h	Cell cycle arrest and apoptosis	MCF‐7	Flow cytometry, qPCR, and Western blot analysis	ZnO‐NPs induce arrest and apoptosis in MCF‐7 cells through several signaling pathways.	^298^
Carbon black nanoparticles	N/A	N/A	N/A	Inflammation and carcinogenesis	N/A	Measurement of polycyclic aromatic hydrocarbon metabolites in urine, a cytokinesis‐block micronucleus assay, and bioinformatics analysis	After exposure to CB, the internal systemic milieu can activate cancer‐related pathways similar but not identical to exposure to the human class 1 carcinogen DEP, increasing lung cancer risk and affecting lung cancer prognosis.	^299^

Abbreviations: NPs, nanoparticles; ELISA, enzyme linked immunosorbent assay; qPCR, real‐time quantitative PCR; ROS, reactiveoxygenspecies.

## 5 EVALUATION MODELS OF NPs

In terms of understanding the toxicity mechanisms of NPs, researchers have found that the toxicity of NPs is closely related to their surface chemistry, shape, size, surface charge, and other factors. In addition, NPs are able to enter the human body through different routes, such as inhalation, oral ingestion, and skin contact, leading to different toxic effects. Therefore, studying the toxicity mechanisms of NPs can help people better understand the toxic effects and hazard mechanisms of NPs, which can guide the safe use and management of NPs. On the other hand, understanding and developing effective models for toxicity evaluation of NPs is also essential to protect human health and the environment. And along with the rapid development and wide application of nanotechnology, the toxicity evaluation of NPs is also receiving increasing attention. In order to better assess the toxicity of NPs, researchers are exploring the toxicity mechanisms of NPs and developing more accurate and reliable toxicity evaluation models, which can help people to better predict the toxic effects of NPs and thus provide a scientific basis for relevant safety assessment and management. Therefore, this section will present the development of toxicity mechanisms and toxicity evaluation models for NPs and discuss the current research progress and future challenges.

The regulations and guidelines related to NP assessment models vary from country to country and agency to agency. The European Chemicals Agency has issued a number of guidelines and recommendations to help companies comply with EU regulations on nanomaterials. These guidelines include regulations for registration, assessment and authorization of nanomaterials. The U.S. Environmental Protection Agency (EPA) has also developed guidelines to help companies and researchers assess the risks and safety of NPs. For example, the EPA provides a NP risk assessment framework that includes assessments of the physical and chemical properties, exposure pathways, bioaccessibility, toxicity, and environmental effects of NPs. And the International Organization for Standardization (ISO) has developed several standards to help researchers assess the risks and safety of NPs. For example, ISO/TS 12901−2:2018 provides guidelines for the evaluation of NP toxicity. It is important to note that these regulations and guidelines are usually advisory, not mandatory.

**FIGURE 3:**
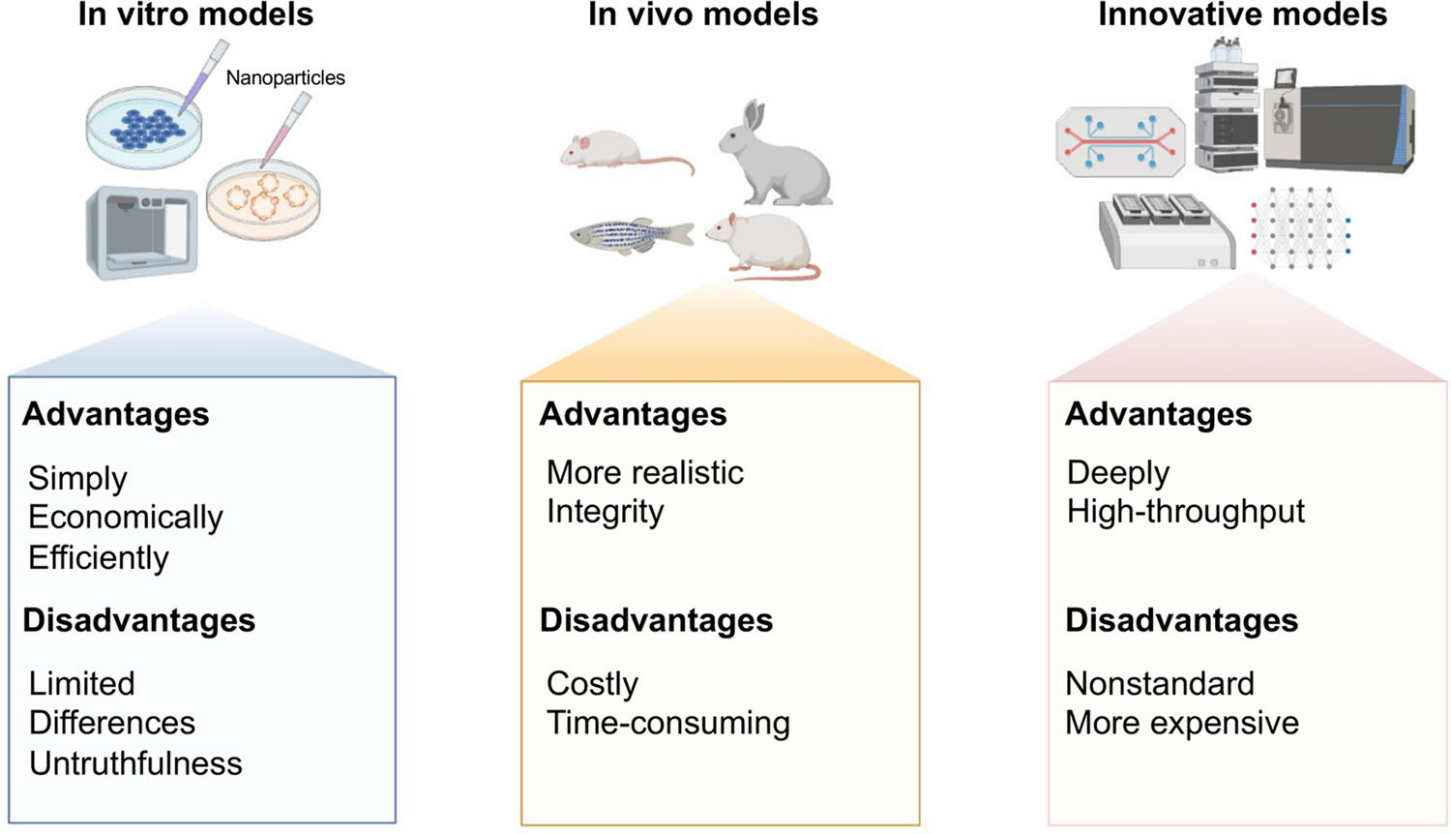
Evaluation models for nanoparticles. A schematic illustration showing in vivo, in vitro, or innovative evaluation models for nanoparticles and the advantages and disadvantages of each evaluation model. The figure elements used was permitted by Biorender (https://biorender.com/).

A variety of specific assessment models have been used in toxicological studies of NPs, including in vivo (represented by small animals), in vitro (2D cells and organoids), and novel models to evaluate the effects and behaviors of NPs^310^ (Table 3 and Figure 3). However, due to the diversity of NPs themselves in terms of structure and properties, as well as the different toxic endpoints observed at different biological levels, the results assessed using different evaluation models are different or even contradictory.^311^, ^312^

**TABLE 3:** Evaluation models of nanoparticles.

Type of NPs	Models/detailed	Size (nm)	Concentration	Damages or treatment	Findings	References
Cu‐NPs	Animal/zebrafish	25 nm	1 mg/L	ROS, inflammation	Promote the transcription of proinflammatory genes.	^310^
PS‐NPs	Animal/rat	38.92 nm	10 mg/kg/day	Nephrotoxicity	Plastic NPs promote endocrine thyroid disorders.	^312^
Single‐walled carbon nanotubes (SWCNTs) and graphene oxide (GO)	Cell/HepG2 cells	1–6 nm	N/A	Oxidative stress and apoptosis	Graphite nanomaterials promote cellular oxidative stress and alter metabolic pathways.	^313^
GO and reduced graphene oxide (rGO)	Cell/HepG2 cells	40 nm	0–200 mg/L	Cytotoxicity, DNA damage, oxidative stress	Different surface oxidation states can lead to different molecular mechanisms of the effect of nanoparticles.	^314^
Au‐NPs	Cell/SK‐BR‐3 cells	15 and 45 nm	1–1000 μg/mL	Cellular adsorption and internalization	Surface functional groups covering the nanoparticles affect the rate of cellular uptake instead of the size of Au‐Nps.	^315^, ^316^
SiO_2_‐NPs	Cell/A549 cells	15 and 46 nm	10–100 μg/mL	Oxidative stress and cytotoxicity	Cytotoxicity of SiO_2_ nanoparticles is dose dependent and associated with oxidative stress.	^317^
Polystyrene NPs	Cell/A549 cells and J774A.1 macrophages	40 nm	20 μg/mL	Endocytosis	The endocytosis uptake mechanism has a significant effect on the cellular uptake of nanoparticles.	^318^
Au‐NPs	Cell/ human dermal fibroblasts	45 and 13 nm	N/A	Cytoskeleton filament disruption and apoptosis	Nanoparticles induce apoptosis and inhibit cell proliferation, but the removal of nanoparticles restores the deleterious effects.	^319^
Au‐NPs	Cell	0.8–15 nm	IC(50): 30−56 mM	Necrosis and apoptosis	Differences in gold nanoparticle‐induced cell patterns are not only related to their size but also to cell type.	^320^
PAMAM‐coated NPs	Cell/Neuro 2A and Vero cells	2.03 nm	0–0.4 mg/mL	Cytotoxicity	Mammalian and microbial cells and different media properties affect the entry of nanoparticles.	^321^
G5‐OH PAMAM	Organoid/kidney organoid	5.2 nm	0–0.9 mg/mL	Nephrotoxicity and cell viability reduced	Exposure to the same nanoparticles can cause nephrotoxicity.	^322^
Multiwalled carbon nanotubes (MWCNT)	3D lung microtissues	75 nm	0.5−10 μg/mL	Increased inflammatory cytokines	Nanoparticles induce differential expression of genes involved in acute inflammation and extracellular matrix remodeling.	^323^
SiO_2_ and TiO_2_‐NPs	Organoid/colon organoid	20 nm	0.8 and 1.1 mM	Cytotoxic	Attention should be paid to inducing cytotoxic effects when applying nanoparticles.	^324^
PS‐NPs	Organoid/intestinal organoids	50 nm	10 and 100 μg/mL	Cell apoptosis and inflammatory response	Inhibition of endocytosis alleviates the toxicity of PS‐NPs to the intestine.	^325^
Ag‐NPs	Organoid/cerebral organoids	20 nm	0.1 or 0.5 μg/mL	Developmental neurotoxic effects	Ag‐NPs (0.5 g/mL) inhibit cell proliferation and induce apoptosis, altering protein expression and ultimately disrupting neuronal growth.	^326^
SiO_2_‐NPs	Animal/BALB/C mice	50 nm	92–114 mg/kg	Systemic toxicity and inflammation	Damage caused by intravenous injection of SiO_2_‐NPs is related to nanoparticle size, and animal sex.	^327^
SiO_2_‐NPs	Animal/BALB/C mice	50 nm	100 mg/kg	Liver inflammation and neutrophil aggregation	Acute histotoxic effects of silica nanoparticles at the maximum tolerated dose take up to 1 year to recover.	^328^
mesoporous silica NPs (MSNs)	Animal/ICR mice	N/A	Intravenous (20 mg/kg/d) or oral administration (200 mg/kg/d)	Hepatic injury and hepatic metabolism function changes	The mode of nanoparticle administration has a significant impact on hepatic metabolism, with oral administration causing less damage.	^329^
mRNA‐loaded dendrimer lipid nanoparticles (mDLNPs)	Animal/FAH^−/−^ knockout mice	N/A	N/A	Restoration of FAH protein function, weight, and liver function	The study presents a modified nanoparticle with the potential to treat hereditary liver diseases.	^330^
Ferritin NPs	Animal/mice	N/A	N/A	Targeting dendritic cells	A platform for ferritin nanoparticle‐based tumor immunotherapy is provided.	^331^
Starch‐coated iron oxide magnetic NPs	Animal/Fischer rats	100 nm	N/A	N/A	Magnetic nanoparticle thermotherapy may be able to treat spinal tumors.	^332^
PS‐NPs	Animal/Wistar male rats	38.92 nm	1–10 mg PS‐NPs/kg/d	Neurobehavioral effects	The uptake of pristine nanoparticles may have a clinically significant impact.	^333^
Carbon‐based NPs	Animal/zebrafish	N/A	5–500 ppm	No adverse effects	Development and research of therapeutic carbon‐based nanomaterials should take into account the differences in different growth stages.	^334^
Carbon‐based NPs	Animal/rabbits	N/A	100 mg/kg			
CPT‐loaded polycationic CD‐NPs	Animal/mice	N/A	5 mg/kg	Colorectal tumor masses and number of liver metastatic foci reduction	Promising tumor treatment with modified nanoparticles.	^335^
Magnetic NPs	Innovative/microfluidic	18 nm	N/A	N/A	Microfluidics allows highly sensitive assessment of cell deformation when in contact with nanoparticles.	^336^
Ag‐NPs	Innovative/microfluidic chip	<100 nm	0.01 mg/L	N/A	Microfluidics can be combined with nematodes for rapid and specific assessment of silver nanoparticle toxicity.	^337^
Ag‐NPs	Innovative/single‐cell RNA‐sequencing (scRNA‐seq)	40 nm	2 μg/mL	Oxidative stress and phagocytosis	The cellular response to nanoparticles is heterogeneous among different cell types, and the tolerated dose varies among different cell types.	^338^
CuO‐NPs	Innovative/untargeted metabolomics	28.2 nm	10 μg/mL	Oxidative stress, hypertonic stress, and apoptosis	Nontargeted metabolomics analysis can be used for NP toxicity screening.	^339^

### 5.1 2D cell models

Normal and cancer cell lines^340^ are often used as models for in vitro studies to assess toxic substances, including NPs, more economically and efficiently.^341^, ^342^ In brief, a variety of cells of interest are exposed to different concentrations of NPs, incubated for different times, and combined with different experimental methods to assess the color and fluorescent change of the exposed cells.^343^

A study investigated the potential cytotoxicity of oxidized single‐walled CNTs and graphene oxide on the liver, a vital organ, using a combined protein profiling approach on human liver cancer HepG2 cells.^313^ HepG2 cells have also been used to study the differential surface functionalization of grapheme nanomaterials.^314^ Using the cancer cell line HeLa cells as a model for coculture with NPs, a comprehensive investigation of the factors influencing cell phagocytosis efficiency and the mechanism of phagocytosis was achieved, providing new ideas and options for the design and development of nanocarrier systems in the biomedical field.^344^ NPs can also interact with cancer cells to provide a framework for high throughput interrogation and improved rational design of nanocarriers.^315^, ^316^ In a study, Au‐NPs were utilized as a SERS substrate to assess the cytotoxicity of TiO_2_‐NPs and single‐walled carbon nanotubes on two different cell lines, A549 (human lung cancer) and HSF (human skin fibroblasts).^345^ In another in vitro study on human bronchoalveolar carcinoma‐derived cells, SNPs were found to increase cytotoxicity in a dose‐dependent manner by a mechanism closely related to oxidative stress.^317^ Kuhn et al.^318^ used a mouse macrophage (J774A.1) and a human alveolar epithelial type II cell line (A549) to study NP uptake pathways and discovered that uptake of 40 nm PS NPs requires more than one endocytic uptake mechanism. More specifically, it has been demonstrated that Au‐NPs can have negative effects on human skin fibroblasts, which are affected by the size, concentration, and duration of exposure, with 45 nm Au‐NPs penetrating cells via lectin‐mediated endocytosis, while smaller NPs enter cells primarily through phagocytosis.^319^ Another study examined the specific mechanism by which lipoic acid‐protected Au‐NPs is taken up by live HeLa cells, a process that requires energy and is eventually transported to lysosomes.^346^ Using cellular models, the researchers found not only that different types of cells are not equally sensitive to NPs, but also that different sizes of NPs lead to cell death in different ways, with smaller NPs leading to faster cell death patterns regardless of cell type.^320^ Changes in ROS levels are typically one of the ways that cytotoxicity expresses itself. The use of HEK293 and LNCaP cells to investigate the significant changes in ROS levels upon exposure to iron oxide NPs is a very rapid evaluation model.^347^ In other investigations, mammalian and microbial cells were combined to examine the toxicity of polyamidoamine (PAMAM)‐coated NPs and the variations in how biological systems are impacted by them.^321^ Human‐derived cells can also be used to assess the immunotoxicity of NPs.^348^, ^349^, ^350^ The immune system can play a role in protecting the body from the threat of foreign NPs, so lymphocytes and macrophages are commonly used to assess the immunotoxicity of NPs. An experiment in lymphocytes revealed that Ag‐NPs ≤ 20 nm in size caused an inflammatory response in lymphocytes and increased the level of ROS.^351^ Another study explored the functional modulation and immunotoxin effects and mechanisms of SNPs on macrophages in mice, which were not only related to the properties of NPs but also the stronger immune response of immune cells.^352^ Hepatocytes that have been extracted and cultivated from sick people are also ideal for testing the toxicity of NPs on the condition of the liver.^353^ Although most studies use cells to evaluate NPs, more attention should be paid to the mode of administration during the experiments, which may have a significant impact on the authenticity and reproducibility of the results, since the exposure process of NPs to cells also involves the stability of NPs and their fluid dynamics.^354^ The direct addition of higher concentrations of NPs to cells has a stronger effect on cells than prediluting and mixing NPs with the medium before adding them to cells, but the mechanisms involved are not obvious, so the evaluation of NPs at the cellular level requires more data to support the view. Due to the limitations of the two‐dimensional cell culture model, one study also compared the difference in toxicity between a three‐dimensional cell model with SNPs cultured in an extracellular matrix and a two‐dimensional HepG2 cell culture model, and in agreement with expectations, the results were different, with more severe damage in the two‐dimensional cell model.^355^

While in vitro cellular assays can help assess the toxicity of nanomaterials, they may not be able to account for the complexities of pharmacokinetics, organ toxicity, and preclinical and clinical conditions. Additionally, the results can differ significantly from study to study and laboratory to laboratory. However, the first step in creating viable methods to prevent nanotoxicity and promote safer use is understanding the molecular causes of nanotoxicity in various cells.

### 5.2 Organoids

Organoids are three‐dimensional aggregates of cells derived from embryonic stem cells, induced pluripotent stem cells, or adult stem cells that replicate multiple physiological and genomic features of various tissues or organs. Organoids provide a more physiological setting for cell‐to‐cell interactions and cellular responses, while retaining certain specific features of the tumor samples from which they originate and allowing for more accurate characterization of different individuals,^356^, ^357^ although they can be technically challenging and have been used more frequently in recent years and have become a much sought‐after in vitro model.^358^

Since the successful establishment of intestinal organs, organs of human origin such as the colon, lungs, and liver^359^, ^360^ have also been created.^361^, ^362^ Astashkina et al.^322^ employed a 3‐D kidney organoid proximal tubule culture to assess the in vitro toxicity of the hydroxylated generation‐5 PAMAM dendrimer (G5‐OH) and Au‐NPs as compared with previously published preclinical in vivo rodent nephrotoxicity data. Kabadi et al.^323^ cocultured human lung fibroblasts, epithelial cells, and macrophages to form scaffold‐free 3D lung microtissues to evaluate nanomaterial‐induced cell–matrix alterations and delineation of toxicity pathways, which was a more predictive and physiologically relevant model for NPs toxicity testing in vitro. Park et al.^324^ assessed the toxicity of silicon dioxide (SiO_2_) and TiO_2_‐NPs in vivo and human colon organoids, and they found that SiO_2_ and TiO_2_ are cytotoxic for human beings. Organoids are used in a variety of areas including personalized drug development, disease modeling precision medicine.^363^, ^364^ Hou et al.^325^ showed that intestine organoids of various cell types accumulate with PS‐NPs(50 nm in size), which causes cell death and inflammatory response. According to Huang et al.,^326^ high concentrations of Ag‐NPs (0.5 g/mL) in cerebral organoids decreased cell proliferation, caused apoptosis, and hindered neurite development, confirming Ag‐NPs are a latent congenital risk factor.

From 2D cultures that reveal the molecular mechanisms of cells to 3D structural reconstructions of human organs, researchers have sought to find more highly realistic model systems that can also be used in a wide range of applications. Although the current organoid system is in its early stages and cannot completely replace all conventional models,^365^ using organoid models in conjunction with conventional models will provide new opportunities for NP evaluation.

### 5.3 Animal models

Assessment data from in vivo models are often more realistic and crucial, but compared with other traditional methods, small animal models are more costly and time consuming.^366^, ^367^ A growing number of studies are being conducted to evaluate NPs using animals as models.

The potential long‐term toxicity of NPs may be related to the sex of the mice,^107^ with males showing a lower tolerance to injected mesoporous SNPs.^327^ A team also evaluated the chronic toxicity of SNPs based on changes in particle size and porosity using the BALB/c mice model, and revealing the potential utility of SNPs in biomedical applications.^328^ Li et al. used a combination of metabolomics with transcriptomics and proteomics to assess the effects of intravenous and oral administration of mesoporous SNPs on liver function and further elucidated the effects of NP exposure patterns on the organism and system using mouse models.^329^ Cheng and Wei showed that therapeutic FAH mRNA delivered by dendrimer‐based lipid NPs normalizes liver function and lengthens survival, via a mouse model of hepatorenal tyrosinemia type I.^330^ In an ototoxic mouse model, researchers discovered that dexamethasone‐loaded PLGA NPs delivered by magnetic attraction can prevent hearing loss.^368^ Using a mouse model of collagen‐induced arthritis and a human transgenic mouse model of arthritis, Zhang et al.^369^ discovered that neutrophil membrane‐coated NPs exhibit significant therapeutic efficacy by reducing joint destruction and the severity of arthritis as a whole. There are also studies that examined the antipsoriatic and toxic effects of methotrexate‐loaded chitin nanogels in an imiquimod (IMQ)‐induced mouse model, and they discovered that they were an ideal delivery platform for topical methotrexate delivery in psoriasis due to their significant antipsoriatic efficacy on IMQ‐induced model of psoriasis without any dermal and systemic toxicities.^370^ Evaluation of antigen‐specific cytotoxic T lymphocyte responses based on the SpyCatcher‐modified ferritin NP platform using a knockout mouse model for personalized tumor immunotherapy.^331^ Rats were used as a model of hypertensive disease to study the pharmacokinetic profile and pharmacodynamic evaluation of nanoemulsions on experimental hypertension.^371^ Using a rat model of metastatic spine disease, the author discovered that locally delivered magnetic NPs activated by an AMF can induce hyperthermia in spinal tumors without harming the spinal cord or accumulating in the lymphoreticular system, thereby limiting neurological dysfunction and reducing systemic exposure.^332^ Rafiee et al.^333^ assessed the neurobehavioral toxicity of rats given pristine PS nanoplastics by oral exposure and found that rats fed with NPs did not detect any significant behavioral changes or abnormality. However, taking into account the subtle and transient nature of neurobehavioral effects, these results highlight the possibility of even pristine plastic NPs induce behavioral alteration in the rest of the food web, including for other animals.^333^ According to Lin and Yen's evaluation of the potential toxicity of therapeutic carbon nanomaterials using tests on various animal models and developmental stages, Lys‐CNGs did not have any negative effects on weight loss, dermal irritation, or skin sensitization responses in rabbits and guinea pigs even at a high dose of 2000 mg/kg body weight.^334^ In an in vivo liver cancer model using rabbits, Glazer et al.^372^ evaluated the acute toxicity and biodistribution of naked Au‐NPs. They discovered that 5 nm Au–NPs were identified in the liver at substantially higher concentrations than 25 nm AuNPs. Despite this, there is little to no indication of acute toxicity and the Au–NPs appear to be well tolerated. Zebrafish, an environmental toxicology model organism, has also been used by many scholars to investigate the biosafety effects of different NPs.^373^, ^374^ Although the complexity of zebrafish physiology is not as close to that of mice and humans, the zebrafish model is superior because of their transparent embryos, short life cycle, and rapid reproduction,^375^ it can economically, efficiently, and with high fidelity meet the requirements for HTS of emerging NPs.^376^ Using the zebrafish model, Teijeiro‐Valio and Yebra‐Pimentel^377^ evaluated the effectiveness of a novel drug delivery nanocapsule with a double shell of hyaluronic acid and protamine and discovered that its toxicity was significantly lower than that of a control nanoemulsion. Using endothelial cells in a zebrafish model to assess the cardiovascular toxicity of SNPs, Duan et al.^335^ found that these particles could interfere with the development of the heart and block angiogenesis. And with the use of a small animal ARDS model, Jani et al.^378^ discovered that NO‐NPs therapy enhanced arterial PO_2_ at high FiO_2_ compared with inhaled NO alone and decreased the number of neutrophils in the circulating and pulmonary interstitial fluid, but inhaled NO did not. Another study discovered that polycationic CD‐NPs can transfer the therapeutic load up to the colon and have a tendency to aggregate particularly in tumor foci, indicating an efficient local therapy method using early and late‐stage colorectal tumor‐bearing animal models.^379^ In addition, animal models provide a more comprehensive assessment of the organ‐targeting mechanisms of NPs.^380^

Animal models are an extremely important experimental method and tool in nano related evaluation research, contributing to a more convenient and effective understanding of NP properties, disease development patterns, and the study of preventive and curative measures.

### 5.4 Human models

Despite the promising potential of human assessment models in evaluating the toxic effects of NPs, the limitations and challenges of their application cannot be ignored. There is currently no single validated evaluation method for predicting potential biological risk levels in humans, as they are usually based on known experimental data or assumptions. The challenge is to validate and standardize these models and to develop reliable and relevant endpoints for toxicity assessment, which requires additional effort and energy. The current lack of adequate data support requires that we be able to experimentally obtain some valuable information for risk analysis in the laboratory rather than conducting large‐scale clinical studies. In addition, the development and implementation of human assessment models is time and resource intensive and may not accurately reflect the complexity of the human body and its response to NPs, an issue that needs to be studied in depth. As a result, there is currently no single validated method that can be used in all situations. The development of a human assessment model for the toxicity of NPs is a critical step in advancing the safe use of these materials. These models can provide a more physiologically relevant context for assessing the potential negative effects of NPs while helping to identify the key factors contributing to their toxicity. As human evaluation models continue to evolve and improve, we will be able to more comprehensively and accurately assess the potential adverse effects that NPs may have on human health.

### 5.5 Innovative models

For a more accurate assessment of NPs, there is a need for more advanced and specific assessment models. The targeting of NPs to various parts of the human body is a complex process,^381^, ^382^ and it is difficult to simulate a realistic scenario of NP action with a single model, and the heterogeneity of samples from different organisms complicates accurate assessment even further. Additionally, the development of contemporary technology and advancements in molecular biology can offer fresh and original approaches to evaluating the toxicological behavior of various NPs.

Microfluidic models have been developed for the evaluation of NPs. Microfluidic technology with precise fluid control, combined with single‐cell culture models,^383^ organ,^384^, ^385^, ^386^ or tumor culture chip models,^387^, ^388^ enables more accurate detection of NP properties, toxicity, and transport,^389^ resulting in a more relevant in vivo evaluation system and more efficient screening of NPs. One study used a microfluidic approach to evaluate the interaction of magnetic NPs with human red blood cell membranes and compared it with traditional hemolysis assays, discovering that this microfluidic device is more sensitive than traditional hematology assays.^336^ Nematode‐conjugated microfluidic microarrays have also been used for NP evaluation, which rapidly and specifically assesses the toxicity of Ag‐NPs by detecting body growth and gene expression.^337^ XCELLigence,^390^ an in vitro noninvasive approach that enables continuous data gathering in real‐time and has been utilized in multiple studies, may successfully remove erroneous results seen in other assay models.^391^, ^392^ The toxicity of several inorganic nanomaterials has been investigated by Scott Boitano's team, and it has been contrasted with conventional techniques.^393^ Additionally, it has been demonstrated that the complementary application of mass spectrometry and scRNA‐seq can aid in shedding light on the complex interactions between immune cells and NPs and can be incorporated into upcoming toxicological evaluations of nanomaterials.^338^ Developments in artificial intelligence^394^ and machine learning have also led to the emergence of more cost‐effective nanotoxicity evaluation models,^395^ such as the computational technique known as quantitative structure–activity relationships or QSAR, pharmacokinetic (PBPK) models.^395^, ^396^ Quantitative structure‐property connections are used to characterize a variety of theoretical modeling approaches used to predict the physiochemical properties of nano molecules.^397^ Multiomics^398^ approaches including genomics, transcriptomics, proteomics, and metabolomics are possible to anticipate new biological pathways that are related to nanotoxicity and to identify cytotoxicity at low exposure levels to NPs.^339^, ^399^

There are still some limitations and challenges with the current models used to assess the toxicity of nanomaterials. For example, organ‐on‐a‐chip technology and 3D cell culture models are more complex and expensive than traditional toxicity testing methods, which may limit their widespread use. And there is a lack of standardization among the various techniques, making it difficult to compare results from different studies and to establish consistent guidelines for assessing the toxicity of NPs. There are also emerging models, such as in silico modeling and HTS, that involve the use of computational models or large numbers of NPs, raising ethical questions about the use of animals and the potential for false positives or false negatives. In addition, some emerging m models may not be applicable to all types of NPs or biological systems, limiting their use in certain situations. Despite these limitations and challenges, emerging nanomaterial toxicity assessment models provide excellent new approaches to improve our understanding of the potential health effects of these materials. Continued research and development is needed to further refine these techniques and address their limitations. Overall, more advanced evaluation models should be created to get beyond the limitations of the current traditional approaches and bridge the gap between the impacts of NP toxicity mechanisms in vitro and in vivo and clinical data.

## 6 CHALLENGES AND PERSPECTIVES

As discussed above and previous studies that nanotechnology has been widely applied in multiple fields including medicine, agriculture and industry. However, due to the potential toxicology effects of nano particles and the limitation of the current study models, we list a number of key scientific issues toward a holistic view of the nano particles toxicology and nanotechnology which may also be the typical concerns in the next 10 years.

1. As described above, toxicological events in response to nano particles are typically complex. There might exist the following conditions, one is that one kind of nano particles may induce multiple body systems damage, another is that multiple kinds of nano particles may lead to the one body system damage or multiple organs damage. This consideration should be paid more attention to the future study.
2. Generally, the underlying mechanism of nano particles on health is very important since the results can help to find the potential targets for prevention and clinical therapy. Although extensive mechanisms study has been done and reported, as the rapid development of many new nano particles formation, there are still a long way to discover the potential mechanisms of nano particles in the future. Look ahead, facing the extremely large dataset outcomes raising for the mass spectrometry and gene sequencing high throughout, the statistical analysis method is required to develop for more efficient prediction and analysis.
3. Dose– and time–response reactions still remain the most basic study for the nano particles study. In particular, the effects of low‐dose or the very low‐dose exposure of nano particles on the health should be concerned. Although low dose effects have been attracted more and more attention over the past decade, the evidence regarding the molecular mechanisms, biomarkers, and the possible of clinical inhibitors development or translation need further investigated and mined.
4. The combination effects of two or multiple nano particles on health is typically an important area and draw more and more attention in clinic and scientific community. Underlying mechanisms regarding how and why the combination offers various responses needs to further study.

Indeed, studying on NPs‐induced potential toxicity on human health in the field of interaction of medicine and technology engineer would dramatically contribute to promotion better application of nanotechnology. More than one century studying on this filed has been bearing plenty fruit that the nanotechnology has been improved gradually. Future, the further study should continue to uncover the underling mechanism of nano particles‐induced toxicology and relative development of effective and new study models, ensuring our knowledge of nanotechnology increasing and eventually, eventually to serve for human health.

## AUTHOR CONTRIBUTION

R. X. H. and P. K. Z. contributed to the study concept and critical design of the study. L. H. X., Z. J., and M. S. went through the literatures and analyzed and interpreted the data. L. H. X. and Z. J. wrote the initial manuscript; R. X. H. critically reviewed and M. S. revised the final manuscript. All the authors read and approved the manuscript. Reproduced images were created by Ruixue Huang and the elements used was permitted by Biorender (https://biorender.com/).

## CONFLICT OF INTEREST STATEMENT

The authors declare no competing financial interests.

## ETHICS STATEMENT

Not applicable.

## Data Availability

Not applicable.
